# Putrescine independent wound response phenotype is produced by ODC-like RNAi in planarians

**DOI:** 10.1038/s41598-017-09567-6

**Published:** 2017-08-29

**Authors:** Lucia Cassella, Alessandra Salvetti, Paola Iacopetti, Chiara Ippolito, Claudio Ghezzani, Gregory Gimenez, Eric Ghigo, Leonardo Rossi

**Affiliations:** 1 Department of Clinical and Experimental Medicine, University of Pisa, via Volta 4, 56126 Pisa, Italy; 2 Otago Genomics & Bioinformatics Facility, Department of Biochemistry, University of Otago, PO Box 56, 710 Cumberland Street, Dunedin, 9054 New Zealand; 3 CNRS UMR 7278, IRD198, INSERM U1095, APHM, Institut Hospitalier Universitaire Méditerranée-Infection, Aix-Marseille Université, 19-21 Bd Jean Moulin, 13385 Marseille Cedex 05, France

## Abstract

Despite increasing evidence indicates polyamines as a convergence point for signaling pathways, including cell growth and differentiation, a unifying concept to interpret their role is still missing. The activity of ornithine decarboxylase (ODC), the rate-limiting enzyme in polyamine biosynthesis, is tightly regulated by a complex molecular machinery, and the demonstration of the existence of multiple ODC paralogs, lacking decarboxylation activity, suggests additional layers of complexity to the intricate ODC regulatory pathway. Because of their extraordinary regenerative abilities and abundance of stem cells, planarians have potential to contribute to our understanding of polyamine function in an *in vivo* context. We undertook a study on ODC function in planarians and we found six planarian ODCs (*ODC1-6*). Five out of six ODC homologs carry substitutions of key aminoacids for enzymatic activity, which makes them theoretically unable to decarboxylate ornithine. Silencing of *ODC5* and *6* produced a complex phenotype, by prompting animals to an aberrant response, following chronic injury without tissue removal. Phenotype is neither rescued by putrescine, nor mimicked by difluoromethylornithine treatment. Moreover, the co-silencing of other genes of the ODC regulatory pathway did not modulate phenotype outcome or severity, thus suggesting that the function/s of these ODC-like proteins might be unrelated to decarboxylase activity and putrescine production.

## Introduction

The role of polyamines has been associated with cell growth, aging, memory performance, neurodegenerative diseases, metabolic disorders and cancer^1–6^. Despite the existence of extensive studies addressing polyamine function, a unifying concept to interpret their molecular role is still missing, and their precise biochemical function remains one of the mysteries of molecular cell biology^7^.

As so many processes are affected by polyamines, their intracellular levels are maintained within a relatively narrow range by a number of factors. This is achieved by regulating the complex polyamine biosynthetic pathway at multiple levels, including transcription, translation, and post-transcriptional modification of polyamine biosynthetic enzymes, as well as polyamine transport^2^. This highly regulated metabolic pathway is primed for rapid changes in response to cellular growth signals, environmental changes, and stress.

ODC, the first rate-limiting enzyme of polyamine biosynthesis, is a typical pyridoxal phosphate-requiring enzyme which removes a CO_2_ group from ornithine to yield putrescine. Putrescine, in turn, can be further processed to yield spermidine and spermine. The decarboxylase reaction is performed in the form of a homodimer and X-ray structure analysis revealed two identical active sites at the dimer interface^8^. ODC activity is regulated by a small protein called antizyme (OAZ). The functional form of OAZ is translated at high levels in response to an increase in cellular polyamines; in this case, OAZ interacts with ODC, disrupts the homodimer, and targets the monomer to degradation by the 26S proteasome, in an ubiquitin-independent manner. As a result, polyamine amount is brought back to the basal level^9, 10^.

Antizyme is itself subjected to regulation by an ODC-related protein termed antizyme inhibitor (AZI). Despite its high homology to ODC, AZI is a distinct protein lacking ornithine decarboxylase activity^11, 12^. The most important feature of AZI is its higher affinity for antizyme compared to that of ODC; this explains the ability of AZI to rescue ODC subunits from interaction with antizyme, thus saving them from degradation^13^.

Focusing on the18 amino acid residues thought to be essential for ODC catalytic activity, Ivanov and coworkers^14^ performed a phylogenetic and sequence analysis of over 200 eukaryotic ODC homologs, revealing the existence of a surprisingly high number of ODC-like sequences that have potentially lost their decarboxylase activity, which makes them similar, at least in this respect, to the known vertebrate AZIs. These putative AZI genes have likely emerged repeatedly during eukaryotic evolution and diversification Moreover, many of them have undergone branch- (subphylum-) specific duplication^14^. In their phylogenetic analysis, they found that many organisms express at least one ODC homolog with a complete set of the 18 key amino acids, together with one or more ODC/AZI-like proteins that show substitutions in at least one key amino acid. Authors proposed that the paralogs with multiple alterations have no decarboxylation activity at all, acting more like an AZI then an ODC. However, since ODC functions as a dimer and the active site is formed in the interface between the two monomers, the authors also proposed one alternative possibility in which some of the inactive paralogs form heterodimers with the enzymatically active paralog, thus inactivating it, which would make them ornithine decarboxylase inhibitors. This would add yet another layer of complexity to the already known multiple layers of ODC regulation.

In this paper, we took advantage of the planarian model organism to dissect the role of different ODC paralogs in complex biological phenomena. Planarians are free-living members of the order Tricladida, phylum Platyhelminthes (flatworms)^15^, and express multiple ODC paralogs, three of them identified in the species *Schmidtea mediterranea* by Ivanov and co-workers^14^. The increasing scientific interest in studying planarians arises from three peculiar characteristics of these animals: extraordinary regenerative abilities, food supply-dependent scale of body size, and great abundance of adult stem cells, called “neoblasts”^16–23^. This incredible feature makes planarians a suitable model system in which to study *in vivo* stem cells, stem cell niches, cell differentiation, tissue morphogenesis and organ development. In addition, the possibility to easily perform loss of function studies by RNAi in planarians makes these animals a suitable model system to study *in vivo* the function of single ODC-like genes.

## Results

### Identification of polyamine metabolic enzymes

We identified six different partial sequences (*DjODC1*, *DjODC* *2*, *DjODC3*, *DjODC* *4*, *DjODC*5 and *DjODC6*), coding for putative ornithine decarboxylase-like proteins in the species *D. japonica*, via bioinformatic analysis (Table S1, panel A). Multialignments of both nucleotide and predicted protein sequences revealed that the six genes were significantly different from each other, as indicated by the percentages of identity and similarity indicated in Table 1. Interproscan analysis of the predicted protein sequences^24^ allowed us to identify each sequence as a member of the Ornithine/DAP/Arg decarboxylase family (IPR000183), Ornithine decarboxylase sub-family (IPR002433). In order to investigate whether the six putative ODC isoforms are conserved in the closely related planarian species *S. mediterranea*, we searched for transcripts annotated as “similar to ornithine decarboxylase” in the *Schmidtea mediterranea* genome database (SmedGD)^25^, and we managed to group the results into five (A to E) classes of sequences, each class representing a *S. mediterranea* homolog of ODC (Table S2). Representative sequences from each class were elongated by searching in the Planmine database^26^. According to the expected value (E-value) resulted from alignments of each *D. japonica* ODC nucleotide sequence, to *S. mediterranea* representative members of class A, B, C, D and E, we managed to match *D. japonica* ODCs with their corresponding putative homologs in *S. mediterranea*. The results obtained are shown in Supplementary Table 1 (panel B). A one-to-one correspondence exists for all ODCs but *Smed-odc* class A, that is equally close to *DjODC1* and *2*. Accordingly, we named the *S. mediterranea* ODCs with the progressive numbers of the corresponding *D. japonica* homologs (*Smed-odc-3 to 6*), except for *S. mediterranea* class A that has been called *Smed-odc-A*. We will refer to as “ODCn” when considering the same ortholog from both *D. japonica* and *S. mediterranea* species (i.e. “ODC5” for both *DjODC5* and *Smed-odc-5*). Finally, we decided to compare the predicted protein sequence of each *D. japonica* and *S. mediterranea* ODC with the protein sequence of human ODC, with respect in particular to the conservation rate of the 18 selected key residues which have been described as critical amino acids for ODC catalytic activity^14^. As shown in Table 2, ODC2-6 have aminoacidic substitutions in at least one of the 18 key amino acids. Strikingly, both ODC5 and ODC6 display substitutions in one out of the two essential amino acids, Cys360Val (ODC5) and Lys69Ser (ODC6).

**Table 1:** Identity (bold) and similarity (Italic) percentages between *D. japonica* ODC putative proteins.

	DjODC1	DjODC2	DjODC3	DjODC4	DjODC5	DjODC6
DjODC1		74%	61%	57%	60%	46%
DjODC2	**63%**		60%	56%	62%	49%
DjODC3	**41%**	**42%**		62%	62%	46%
DjODC4	**38%**	**36%**	**43%**		55%	46%
DjODC5	**40%**	**41%**	**39%**	**37%**		53%
DjODC6	**29%**	**27%**	**29%**	**29%**	**33%**

**Table 2:** Alterations of the 18 key aminoacids for ornithine decarboxylase (the numbering of human ODC1 is used). Principal aminoacids involved in catalytic activity are indicated in bold. — = conserved aminoacids; § = missing data sequence.

	K69	D88	R154	K169	G171	H197	S200	G235	G236	G237	E274	R277	C360	D361	D364	G387	Y389	F397
SMED-ODC-A	—	—	—	—	—	—	—	—	—	—	—	—	—	—	—	—	—	—
DjODC2	—	—	—	—	—	—	—	—	—	—	—	—	—	—	—	—	—	—
DjODC1	—	—	—	R	—	—	—	—	—	—	—	—	—	—	—	—	—	—
SMED-ODC-3	—	—	—	—	—	—	N	—	—	—	—	T	—	—	—	—	—	—
DjODC3	—	—	—	—	—	—	N	—	—	—	—	T	—	—	—	—	—	—
SMED-ODC-4	—	—	—	—	—	—	I	—	—	—	T	—	—	—	—	—	—	—
DjODC4	—	—	—	—	—	—	I	—	—	—	T	—	—	—	—	—	—	—
SMED-ODC-5	—	—	—	—	—	—	—	—	—	—	—	A	V	—	—	—	—	—
DjODC5	—	—	—	—	—	—	—	—	—	—	—	A	§	§	§	§	§	§
SMED-ODC-6	S	S	A	—	A	F	T	—	D	—	Q	K	—	—	—	—	—	—
DjODC6	S	S	A	—	A	Y	T	—	D	—	Q	K	—	—	—	—	—	—

In our search, we also identified in *D. japonica* EST collection a single sequence (FY937407.1), called *DjOAZ*, homologous to ornithine decarboxylase antizyme (OAZ) (best blast hit at aminoacidic level with: putative Ornithine decarboxylase antizyme [*Daphnia magna*] 4e^−08^), and a single antizyme sequence (dd_Smed_v6_425_0_1) in *S. mediterranea* Planmine database^26^. For *DjOAZ* an *in-silico* analysis of the putative ORFs was undertaken. We managed to find the peculiar ORF1 and ORF2 distinguishing antizyme proteins (ref. 27, and references therein) (Supplementary Figure 1A); the site of +1 frameshift was characterized by the sequence AUU-UGA-UGA-G (Ile-Stop-Stop), which produced a shift of the reading frame, resulting in AUU-GAU-GAG (Ile-Asp-Glu), allowing the synthesis of the full-length protein. In addition, we also identified a distinctive mRNA secondary structure (hairpin) downstream of the frameshift site, which showed similar features to those of the human OAZ (Supplementary Figure 1B,C).

### Expression of polyamine metabolism enzymes in intact planarians

**Figure 1:**
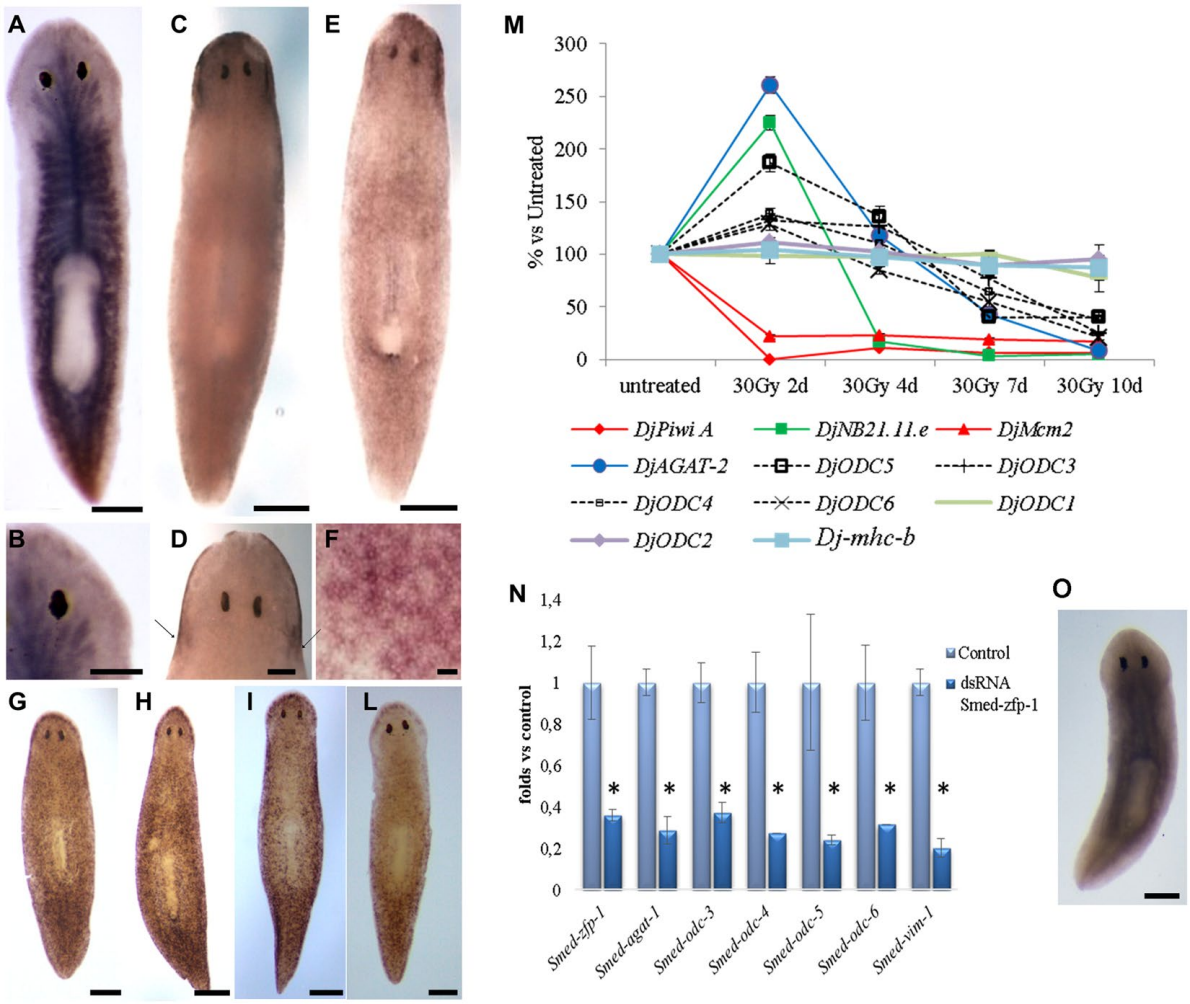
Analysis of ODC and OAZ transcript expression in intact planarians. (**A**) Representative image of an intact planaria hybridized with *DjODC1*. (**B**) Magnification of the head region of an intact planaria hybridized with *DjODC1* probe, dorsal view. (**C**) Representative image of an intact planaria hybridized with *DjODC2* probe, dorsal view. (**D**) Magnification of the head region of an intact planaria hybridized with *DjODC2* probe, dorsal view. Arrows indicate the auricles. (**E**) Representative image of an intact planaria hybridized with *DjODC2* probe, ventral view. (**F**) Magnification of *DjODC2*-positive cells, ventral. (**G–L**) Representative images of intact planarians hybridized with *DjODC3*, *4*, *5*, *6* probes respectively. Scale bars correspond to: 250 µm for (**A,C,E,G–L,O)**; 100 µm for (**B** and **D)**; 50 µm for (**F)**. (**M**) relative expression level of *D. japonica ODC* transcripts and stem cell and stem cell progeny markers in 30 Gy-treated and untreated samples. The trend of progressive decrease in expression of *DjODC3-6* resembles that of *DjAGAT2*, the *D. japonica* homolog of the *Smed-agat-2*. As a comparison, we included in the analysis the *D. japonica* homolog of the *S. mediterranea* early neoblast progeny marker *Smed-NB21.11.e* ^28^ whose expression starts decreasing at an earlier time point (approximately 4d after treatment), in contrast with the markers of proliferating cells *DjPiwiA* ^51^ and *DjMcm2* ^48^ whose signal disappears 24 h after 30 Gy X-ray exposure. *Djmhc-b*, a marker of differentiated sub-epidermal muscle cells^52^, was included as a control for differentiated cells. Each point is the mean ± s.d. of three independent samples, normalized versus the corresponding control, to which an arbitrary value of 100% was attributed. (**M**) Relative expression levels of *S. mediterranea ODC*, *Smed-zfp-1*, *Smed-agat-1* and *Smed-vim-1* (epidermal marker) transcripts, in *Smed-zfp-1* RNAi animals and controls. Each bar is the mean ± s.d. of three independent samples, normalized versus the corresponding control, to which an arbitrary value of 1 was attributed. *p < 0.01. (**N**) representative image of an intact planaria, hybridized with *DjOAZ* probe, dorsal view. Scale bar corresponds to 250 µm.

The analysis of ODC transcript distribution in intact animals revealed a surprisingly complex scenario of expression patterns, involving different types of tissues. *DjODC1* was found predominantly accumulated in the gut (Fig. 1A,B). However, a low widespread expression was observable in several body tissues. *DjODC2* was expressed in the epidermis, with a homogeneous distribution on the ventral surface, and confined to the head on the dorsal surface, with a particularly intense signal in the auricle area (Fig. 1B–D). Magnification of *DjODC2* staining on the ventral surface shows that most epidermal cells are labelled, yet at different level (Fig. 1E). *DjODC3*, *4*, *5*, and 6 shared similar expression patterns (Fig. 1F–I), being expressed in small cells spread in the parenchyma, both anterior to the photoreceptors, and in the periphery of the animal along the anteroposterior and dorsoventral axes. This expression pattern is apparently very similar to that of late epidermal committed neoblast progeny markers (Category 3 genes)^28^.

In order to assess the degree of differentiation of the cells expressing *DjODC1-6*, we analysed the expression levels of these genes by Real Time RT-PCR in intact 30 Gy-treated animals, which were sacrificed 2, 4, 7 and 10 days after X-irradiation. X-ray irradiation in planarians results in rapid and selective elimination of neoblasts, with differentiated cells remaining unaffected at the same time and dose^29^. After irradiation, the expression levels of *DjODC1* and *DjODC2* remained unchanged during the whole time-course (Fig. 1L), demonstrating that these genes are expressed in late neoblast progeny. On the contrary, the expression levels of *DjODC3*, *4*, *5*, and *6*, decreased progressively starting 7 days after irradiation (Fig. 1L). The recent analysis of single planarian cell transcriptome^30^, and the availability of Planarian SCS database (https://radiant.wi.mit.edu/) allowed us to verify that *Smed-odc-3/4/5* and *6* are all expressed in late epidermal-committed neoblast progeny. In addition, we found that expression of *Smed-odc-3 -6* was dramatically reduced in *Smed-zfp-1* RNAi animals (Fig. 1M), in which the formation of z-neoblast, the epidermal lineage precursors, is impaired^22^. This undoubtedly locates *Smed-odc-3 -6* expression in epidermal precursors cells.

*DjOAZ* has a very wide expression pattern (Fig. 1N), consistent with that reported in Planaria SCS @ The Whitehead Institute database for its *S. mediterranea* homolog, suggesting an almost ubiquitous expression.

### Macroscopic effect of *DjODC*s silencing by dsRNA injection

**Figure 2:**
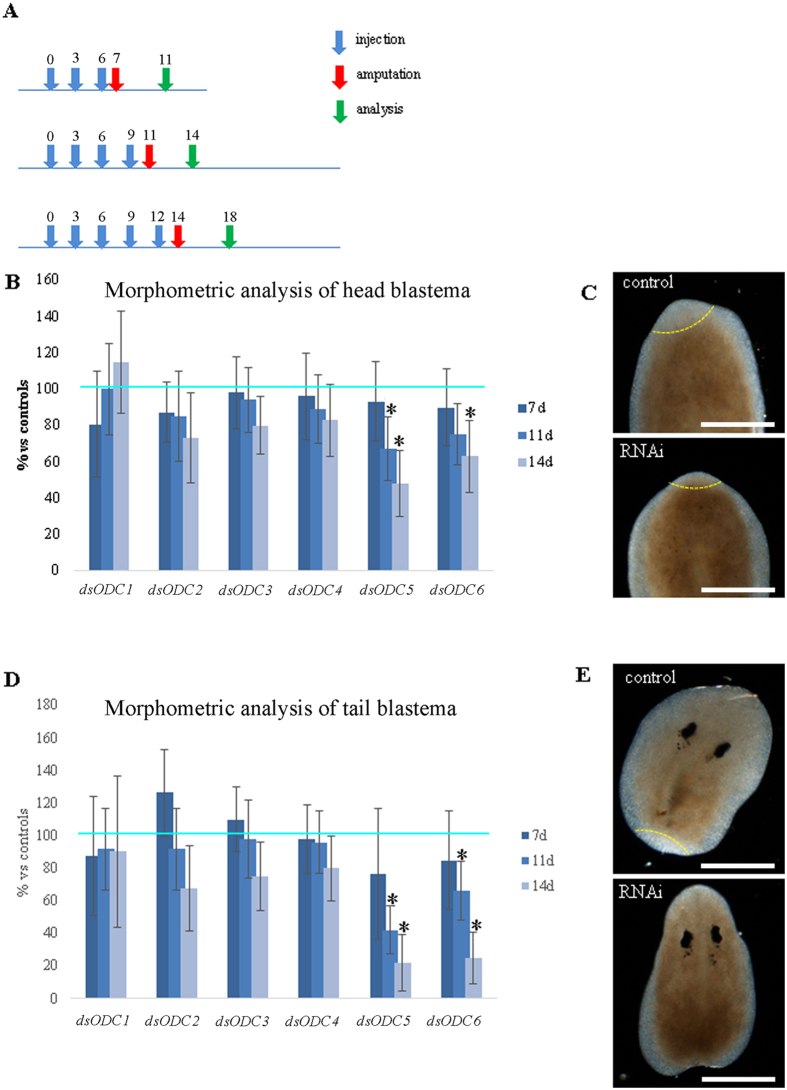
Morphometric analysis of head and tail blastemas after ODC RNAi. (**A**) Schematic representation of the experimental set-up. Numbers indicate the day. Amputation was performed in the neck region. Regeneration rate was assessed by morphometric analysis cutting the animals in the neck region 7, 11 and 14 days after the first dsRNA injection and analyzing blastema size 4 days after the cut. (**B**) Morphometric analysis of head blastemas. Each bar is the mean ± s.d. of two independent experiments (each including 15 different specimens), normalized versus the corresponding control, to which an arbitrary value of 100% was attributed. Light blue line indicates the level of controls. Asterisks indicate cases in which a p value below 0.01 was obtained comparing areas measured in control and RNAi animals in both independent experiments. (**C**) Representative images of planarian fragments regenerating a new head 4 days after cut. Blastemal region is marked by a dotted yellow line. (**D**) Morphometric analysis of tail blastemas. Each bar is the mean ± s.d. of two independent experiments (each including 15 different specimens), normalized versus the corresponding control, to which an arbitrary value of 100% was attributed. Light blue line indicates the level of controls. Asterisks indicate cases in which a p value below 0.01 was obtained comparing areas measured in control and RNAi animals in both independent experiments. (**E**) Representative images of planarian fragments regenerating a new tail, 4 days after cut. Blastemal region is marked by a dotted yellow line. Scale bar corresponds to 250 μm.

**Figure 3:**
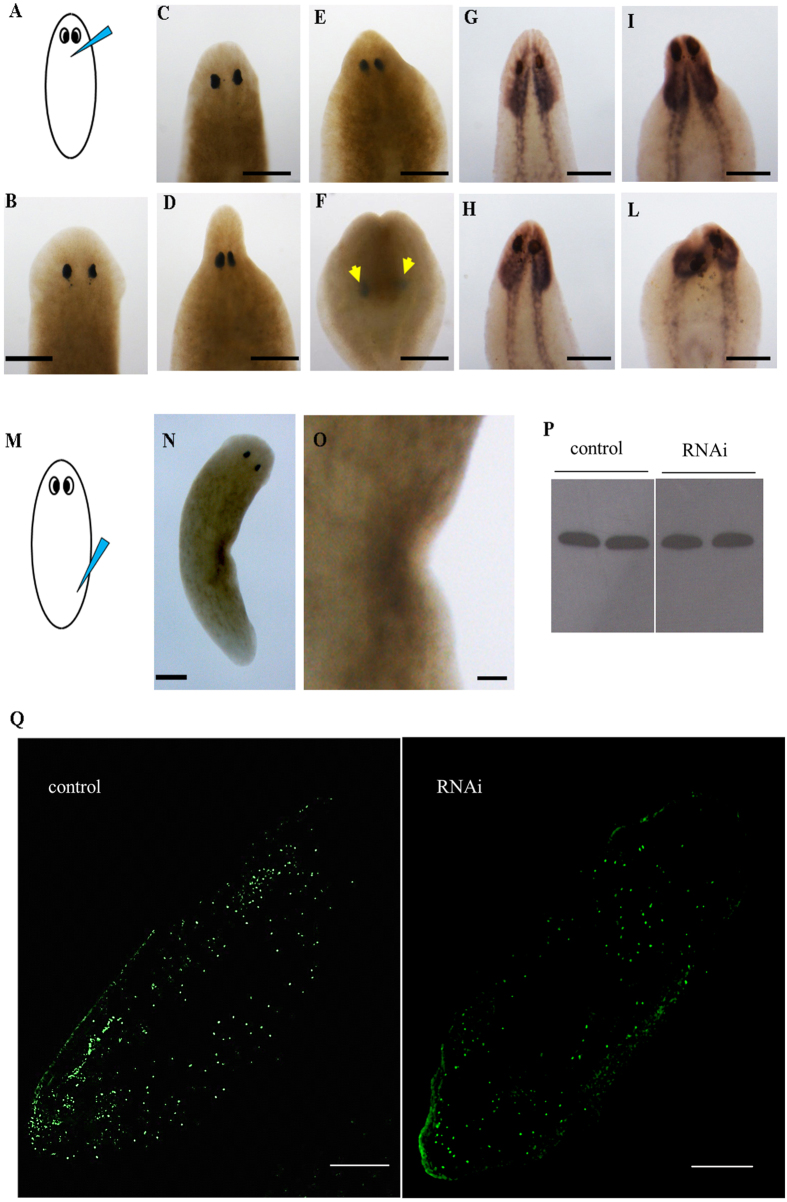
*DjODC5* and *6* (RNAi) macroscopic phenotype and analysis of mitosis. (**A**) Scheme depicting the injection site. (**B**) An intact water-injected control planaria. (**C–E**) *DjODC5* RNAi altered phenotypes, with different degrees of severity. (**F**) The “blemmy” phenotype. Arrows indicate the eyes. (**G–L**) The dynamic movement of head retraction during different stages of phenotype severity can be followed by highlighting the central nervous system with the *DjSyt* marker^53^. (**G**) Whole mount *in situ* hybridization of *DjSyt* in an intact control planaria. (**H**–**L**) Whole mount *in situ* hybridization of *DjSyt* in *DjODC5* (RNAi) phenotypes. Scale bars correspond to 250 µm. (**M**) Scheme depicting the injection site for the phenotype showed in (**N**). (**N**) Phenotype produced by the injection in a posterior gut branch. (**O**) Magnification of N. Scale bars correspond to 250 µm in N and 65 µm in (**O**). (**P,Q**) Immunostaining with anti-H3P. (**P**) Representative Western blot showing a single cross-reactive band for H3P in control and RNAi planarians, injected with *DjODC5* dsRNA molecules in the neck region. (**Q**) Representative images of whole planarians, immunostained with anti-H3P. Number of mitosis/mm^2^ was 153 ± 58, 144 ± 52 and 147 ± 52 in control, *DjODC5* and *6* (RNAi) animals respectively. Scale bars correspond to 250 µm.

As ODC is the rate limiting enzyme in polyamine biosynthesis, we decided to knock down the expression of each of these genes individually by RNAi, to better dissect their roles. dsRNA molecules are normally delivered to planarians by mixing them with liver paste and feeding. However, as liver contains high levels of polyamines, we decided to perform RNAi on both intact and regenerating planarians by injecting dsRNAs. Injections were performed in the anterior gut branch by punching the animals in the space between the eyes and the pharynx, a region that here will be referred to as “neck”. The effect of gene silencing was investigated at different time points until day 21 after the first injection; regeneration rate was assessed by morphometric analysis in animals cut in the neck region 7, 11 and 14 days after the first dsRNA injection (Fig. 2A). *DjODC1*, *DjODC2*, *DjODC3* and *DjODC4* RNAi animals were not affected by gene silencing, as both the intact and the regenerating individuals showed no substantial difference compared to controls (Fig. 2B,D). In contrast, *DjODC5* and *DjODC6* gene silencing caused deleterious effects in both intact and regenerating animals. Eleven days after the first dsRNA injection, both head and tail fragments of *DjODC5* RNAi animals showed severe delays in the regeneration process. Fourteen days after the first dsRNA injection, *DjODC5* RNAi animals produced small sized blastemas, less than half compared to controls; blastema size was particularly reduced in tail fragments (Fig. 2B–E). In some cases (see below), the head fragments died after the cut. *DjODC6* RNAi animals showed a statistically significant delay in regeneration (including some heads’ death after the cut) at this time point as well (Fig. 2B–E). Co-silencing of both ODC genes did not produce a worsening of the regeneration delay (data not shown). After 4/5 dsRNA injections, intact *DjODC5* and *DjODC6* RNAi animals acquired a peculiar phenotype, marked by a progressive reduction of head size, with an evident disappearance of auricles protrusions (Fig. 3C,H), and constriction of the neck region. Later on, the head firstly became roundish, then it was retracted backwards into the “neck” region (Fig. 3D,E,I,L). These particular morphological changes were displayed by 100% of *DjODC5* RNAi intact animals (30/30). In rare cases (5%), head was eventually completely enclosed into the region anterior to the pharynx (Fig. 3F). This morphological change resulted in apparently headless animals, yet having the eyes still visible through the trunk, a unique phenotype that we called “blemmy”. A similar phenotype was observed in *DjODC6* RNAi intact animals (20/30 animals). Co-silencing of both ODC genes did not produce a worsening of the head regression phenotype. We verified the effectiveness and specificity of gene silencing 7 days after the first injection by Real-Time RT-PCR. A strong reduction of both *DjODC5* and *DjODC6* transcript levels was obtained, following dsRNA injection, and no cross-silencing was observed for other ODC transcripts (data not shown). Once established, blemmy phenotypes survived up to about 15 days, and then died. Supplementary figure 2 shows histological analysis of a representative blemmy phenotype, revealing that the enclosed head was surrounded outwardly by parenchymal tissue near the site of retraction. Head retraction and the blemmy phenotype were specifically obtained when injections were performed in the neck region. Surprisingly, when injections were performed in one of the posterior gut branches, we observed thickening of the tail, followed by constriction towards the injection site, to produce arched planarians: the phenotype was therefore injection site-dependent (Fig. 3M,N). An injection-site directed impairment was also evident when the death rate of amputated fragments, subjected to injection in different body regions, was analysed. As shown in Supplementary Figure 3, a higher number of dead fragments was detected when the cut was performed in the same region of injection, while only sporadic dead fragments were found in control planarians injected with water. A similar analysis performed in *S. mediterranea* specimens revealed an identical scenario, in which only *Smed-odc-5* and 6 produced a site injection-dependent “blemmy-like” phenotype (Supplementary Figure 4A–C).

### Analysis of cell proliferation and death

**Figure 4:**
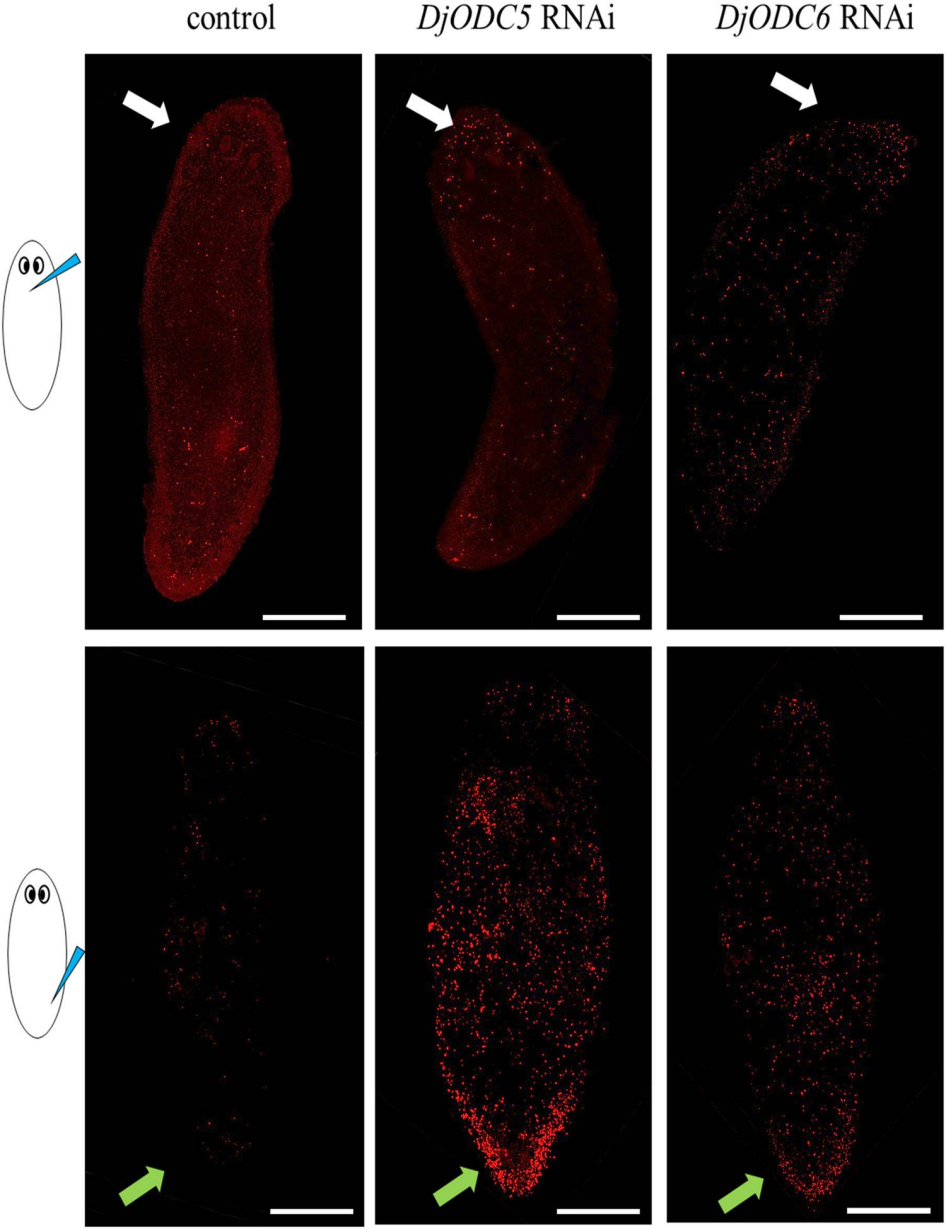
Tunel assay in injected planarians. Tunel positive cells are visualized in red. White arrows indicate the anterior body region. Green arrows indicate the posterior body region. Site of injection is drawn on the left. Scale bar corresponds to 250 µm.

To understand the cellular events underlying this peculiar phenotype, we decided to evaluate cell proliferation and apoptosis rates. The amount of mitotic cells was unaltered in RNAi planarians, with respect to controls (Fig. 3P,Q). Strikingly, RNAi animals showed a significant increase of apoptotic cells, visualized by TUNEL reaction. Apoptosis activation was systemic, but TUNEL-positive cells were particularly concentrated in the body region where dsRNA was injected (Fig. 4). A similar activation of apoptotic response was also demonstrated in *S. mediterranea* specimes (Supplementary Figure 4D). Consistent with the activation of apoptosis, the expression of *Smed-JNK* ^31^ was significantly (p < 0.05) increased in RNAi-treated animals (1,8 ± 0.1 and 1,6 ± 0.08 folds *vs* control in *Smed-odc-5* and *Smed-odc-6* RNAi animals, respectively).

### Histological and ultrastructural analysis

**Figure 5:**
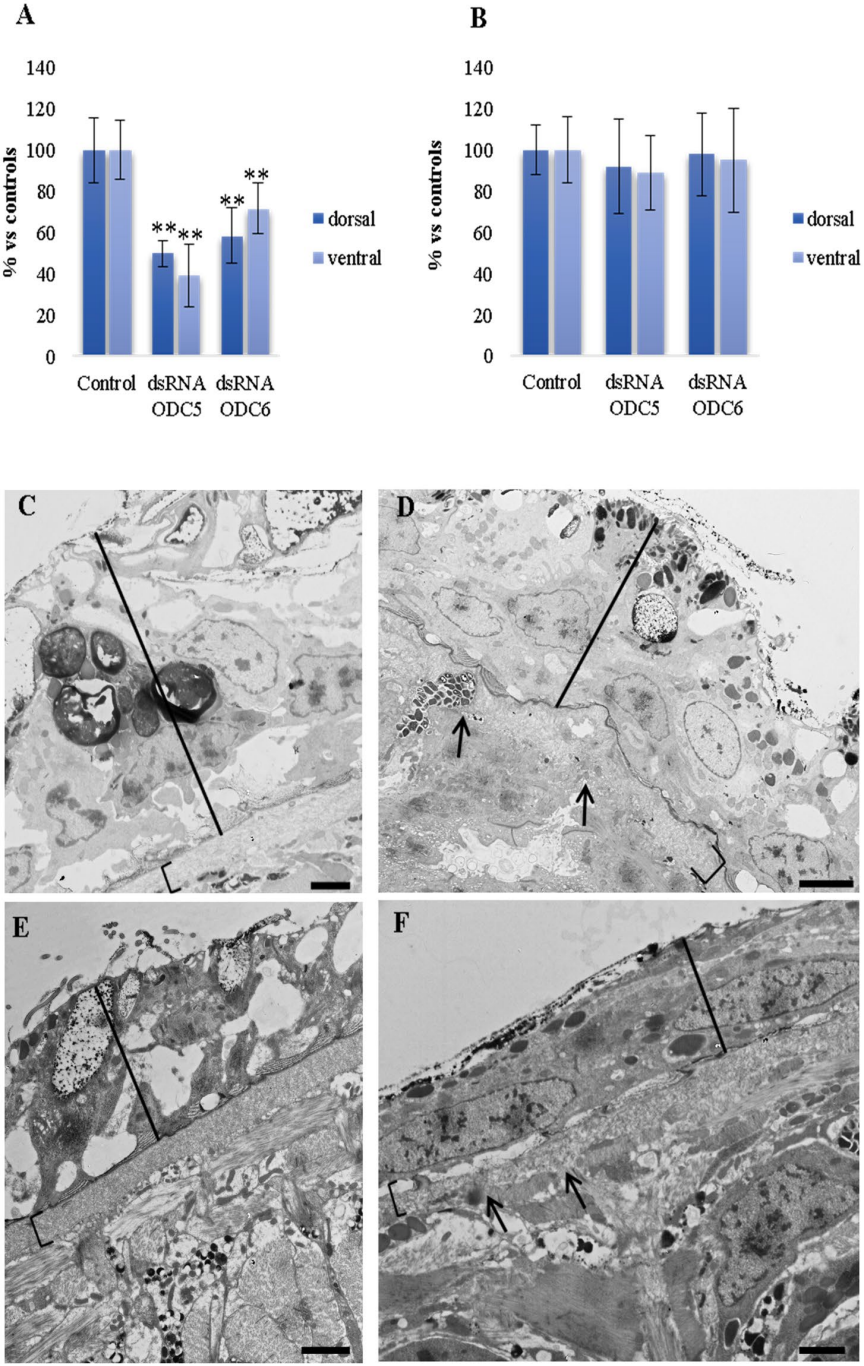
Analysis of epidermal thickness and ultrastructure (**A**) Graph depicting the epidermis thickness evaluated in the body region close to the injection site. (**B**) Graph depicting epidermis thickness evaluated in the body region far from injection site. Each bar is the mean ± s.d. of five independent samples in which epidermis thickness was evaluated in 5 different sections. In each section 6 measurement were taken. Values were normalized versus the corresponding control, to which an arbitrary value of 100% was attributed. **p < 0,001. (**C**) Dorsal epidermis of a water injected control from a body region close to the injection site. (**D**) Dorsal epidermis of a RNAi animal from a body region close to the injection site (**E**) Ventral epidermis of a water injected control from a body region close to the injection site. (**F**) Ventral epidermis of a RNAi animal from a body region close to the injection site. Scale bars correspond to 2 µm. Ultrastructural observations were performed in three independent experiments in which we analyzed several ultrathin sections of two animals per experimental class.

**Figure 6:**
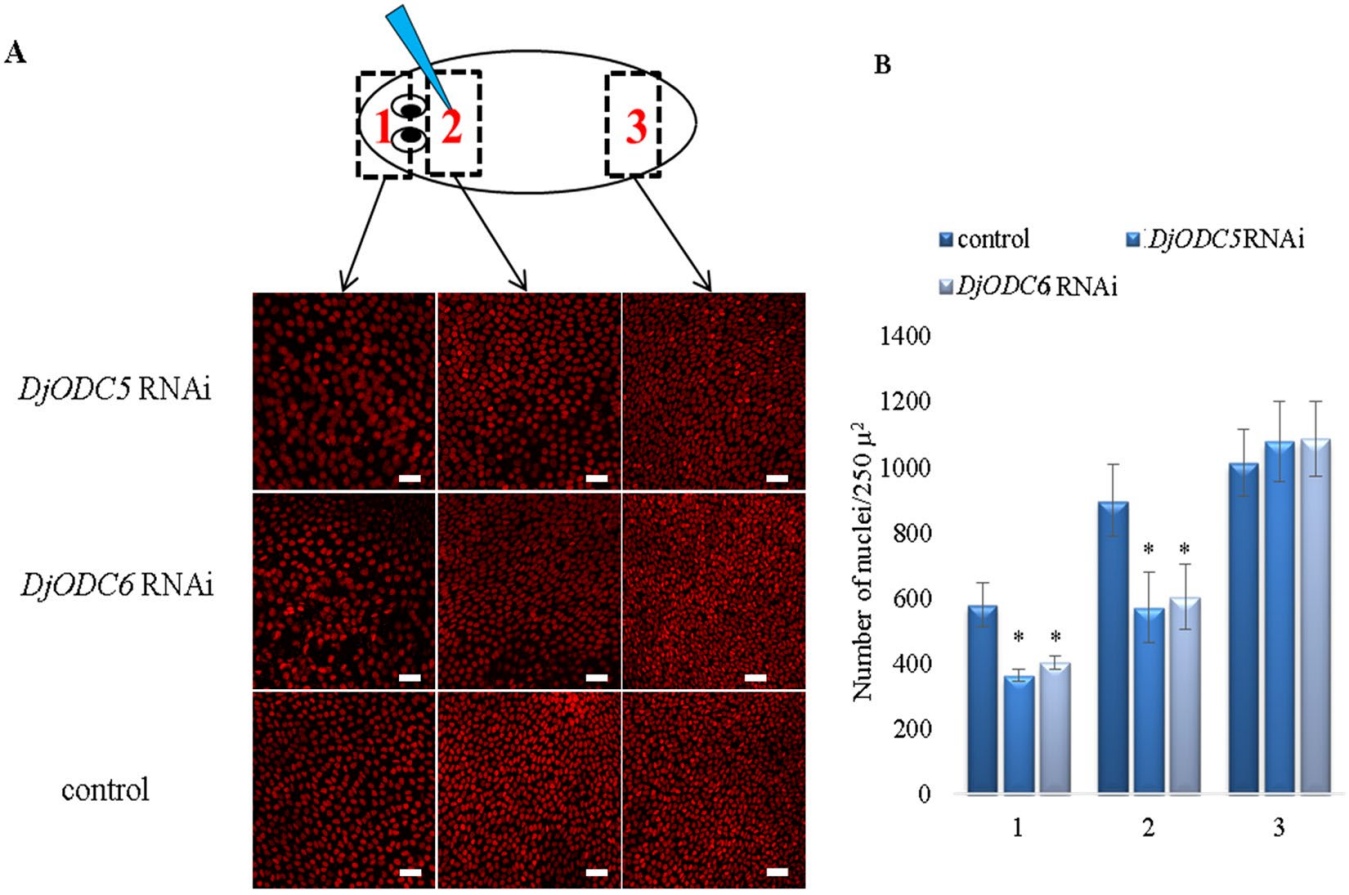
Analysis of epidermis nuclear density in injected organisms. (**A**) Representative images of epidermis nuclei in 3 different body regions^1–3^. Scale bar corresponds to 30 μm. (**B**) Graph depicting numbers of nuclei counted in the different body region of *DjODC5* RNAi, *DjODC6* RNAi and controls. Each bar is the mean ± s.d. of 3 independent samples. *p < 0.01.

**Figure 7:**
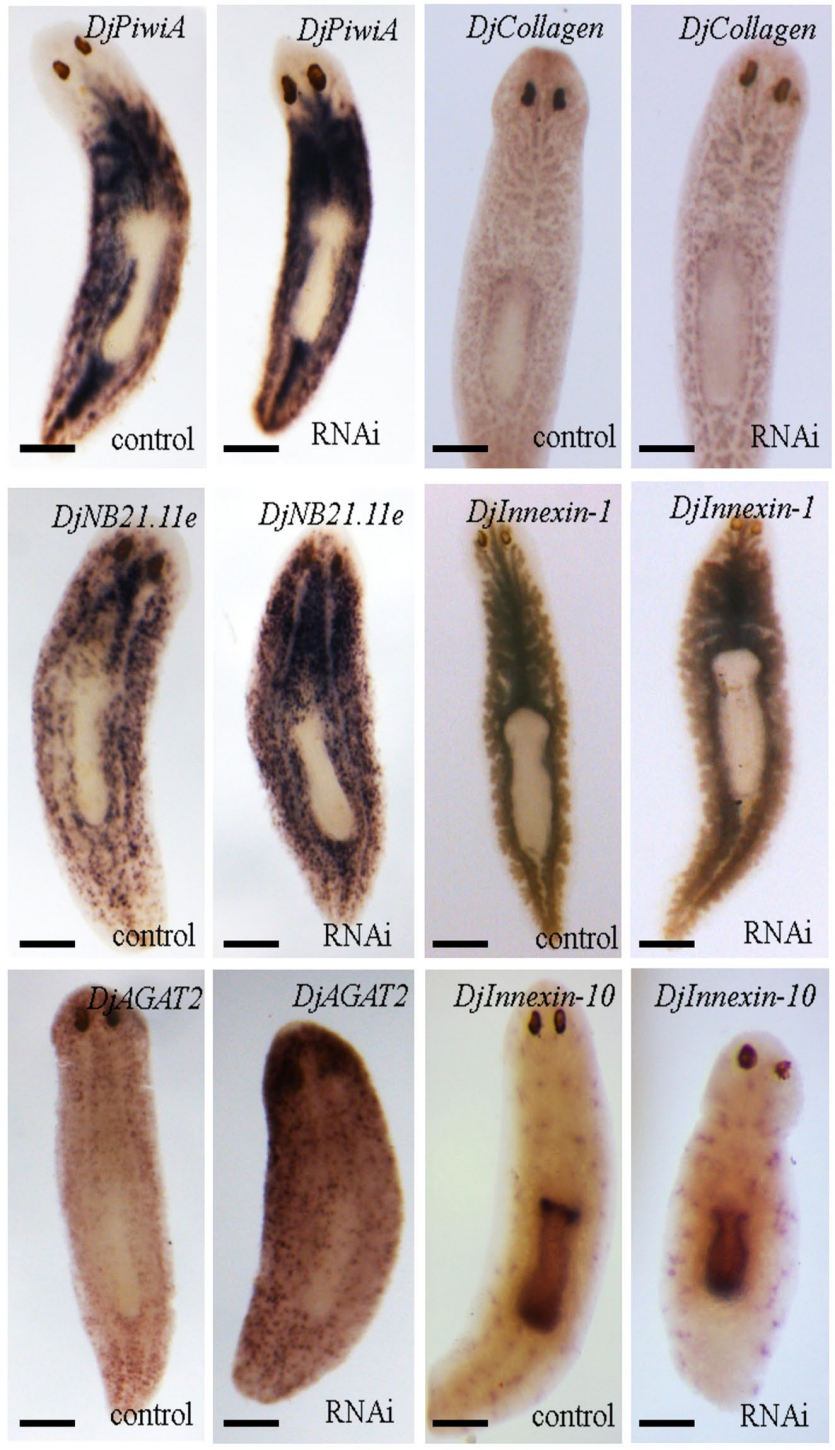
Whole mount *in situ* hybridization of *DjPiwiA*, *DjNB21.11e*, *DjAGAT2*, *DjInnexin1*, *DjInnexin10*, *DjCollagen* on *DjODC5* RNAi animals and controls (analogous results were obtained in *DjODC6* RNAi animals). Scale bar corresponds to 250 µm.

To better understand the effects of gene silencing on planarian tissue organization, we decided to perform morphological investigation of the principal planarian tissue/organs, in animals injected with dsRNA molecules in the neck region. Histological staining of *D. japonica* tissue sections revealed important structural alterations in epidermis, namely a reduction of thickness, a lower density of cilia, and an impairment of apical specialization in RNAi animals, with respect to controls (Fig. 5A,B and Supplementary Figure 5A). These observations were confirmed by ultrastructural analysis (Fig. 5C,F and Supplementary Figure 5B,C). Moreover, epidermal cells appeared disorganized, with changes in nuclear shape and localization. Basal lamina was highly disorganized, thinner (Fig. 5D,F), and in some cases absent. A reduction of epidermal nuclei density was also evident, although not as prominent as alterations in cell shape and ultrastructure (Fig. 6A,B). These effects were injection site-dependent, being maximal when injections were performed in the head region. Similar results were obtained in *S. mediterranea* (Supplementary figure 4E–G). With the aim to investigate possible alterations in other tissues, we decided to analyze the expression of specific tissue markers. Stem cells, early epidermal-committed stem cell progeny, late epidermal-committed stem cell progeny, nervous system, gut and protonephridia, were visualized by whole mount *in situ* hybridization using as probes *DjPiwiA*, *DjNB21.11e*, *DjAGAT2*, *DjSyt*, *DjInnexin1* and *DjInnexin 10* ^32^, respectively. Muscles were visualized using the *D*. *japonica* homolog of the *S*. *mediterranea* general muscle marker *Smed-collagen* ^33^, here called *DjCollagen*. According to what observed above for the central nervous system marker (Fig. 3G–L), and to data obtained with the other markers (Fig. 7 for *DjODC5* RNAi, data not shown for *DjODC6* RNAi), we concluded that the morphology of protonephridia, gut, nervous system and muscle cells was not affected by RNAi, with the exception of structural reorganization due to head contraction. Conversely, either the expression level or the number of positive cells, for early and late stem cell progeny markers, was significantly increased, and a slight increase was detected in stem cell marker expression (Fig. 7). The changes of the expression level of both *DjNB21.11e* and *DjAGAT2* were homogeneous along the animal body and were confirmed by Real Time RT-PCR analysis (*DjNB21.11e* was 3.99 ± 0.98 folds in *DjODC5* RNAi *vs* control, p = 0.02, and 2.09 ± 0.35 folds in *DjODC6* RNAi *vs* control, p = 0.051; *DjAGAT2* was 5.06 ± 0.62 folds in *DjODC5* RNAi *vs* control, p = 0.003, and 2.4 ± 0.48 folds in *DjODC6* RNAi *vs* control, p = 0.048). Real Time RT-PCR analysis of specific tissue markers in *S. mediterranea*, confirmed the results obtained in *D. japonica* specimens (Supplementary Figure 4H).

### ODC5/ODC6 double *in situ* hybridization

**Figure 8:**
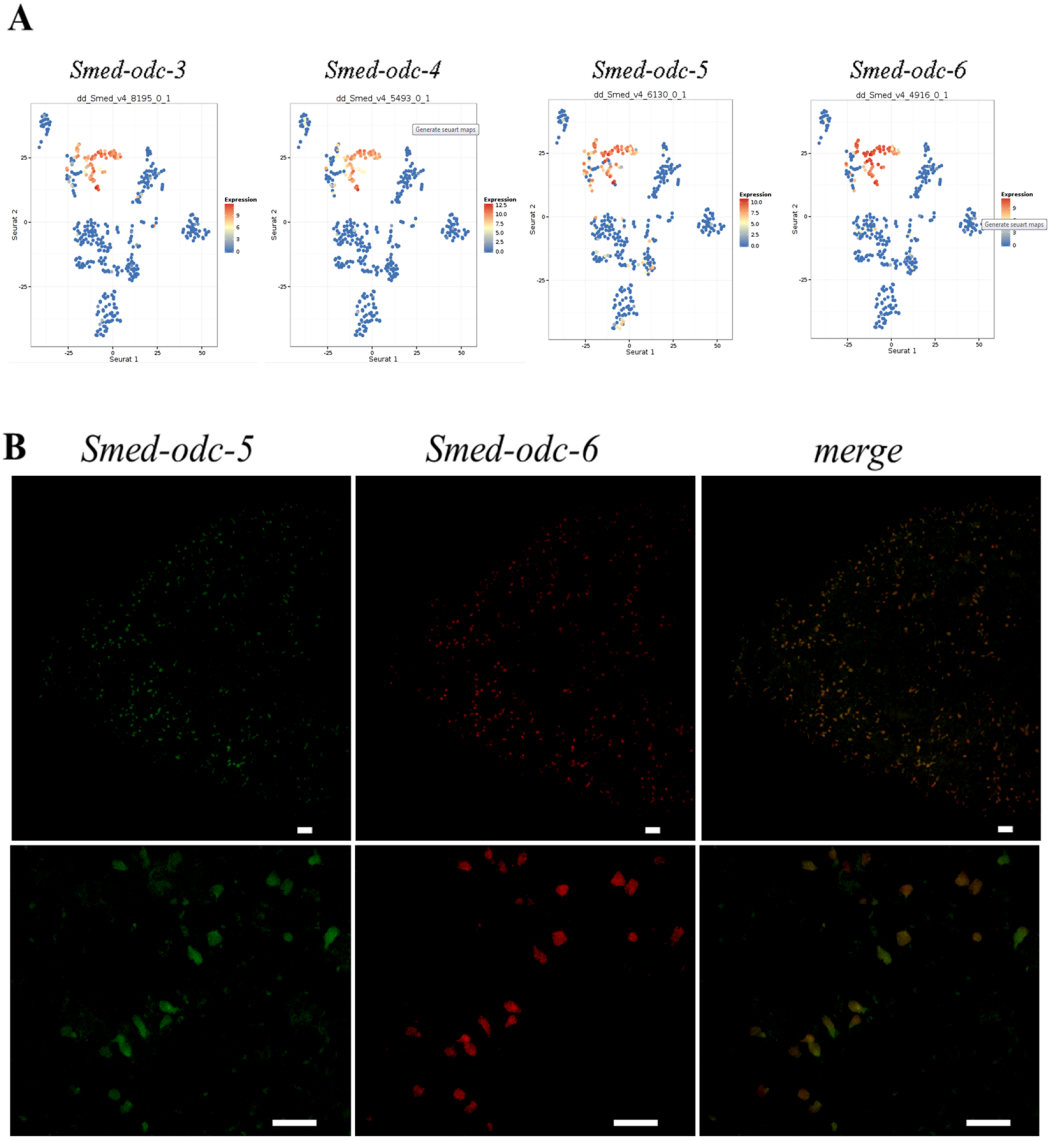
Smed-odcs co-localization analysis. (**A**) Seurat maps generated in Planarian SCS database (https://radiant.wi.mit.edu/)^29^. Each graph represents a t-SNE embedding of single cells based on gene expression. Each cell is represented by a dot. Dot color is attributed according to the expression level of the queried gene (red for maximal expression blue for minimal expression). (**B**) Double fluorescent *in situ* hybridization of *Smed-odc-5* and *Smed-odc-6* transcripts. Scale bars correspond to 25 μm.

According to Seurat maps generated in Planarian SCS database^30^ a large number of epidermal and late epidermal progeny cells share the expression of *Smed-odc-3/4/5/6* (Fig. 8A). To confirm *Smed-odc-5* and *Smed-odc-6* co-localization, we performed expression analysis by double fluorescent RNA *in situ* hybridization. In our experiments *Smed-odc-5* and *Smed-odc-6* were almost completely colocalized, as 99% of analyzed cells were positive for both transcripts (Fig. 8B).

### Effect of *DjODC*s silencing by dsRNA feeding

Some aspects of ODC5 and ODC6 RNAi phenotype are clearly the consequence of the synergistic effect of both ODC silencing and the chronic injuries (without tissue removal) produced by the repetitive injection in a specific body region. To identify which phenotype features are the exclusive consequence of ODC silencing and those that are due to knock-down and chronic punching we decided to deliver dsRNA molecules by feeding using egg yolk, which is known to contain low amounts of polyamines^34^, rather than liver paste as food. *D. japonica* and *S. mediterranea* were fed two times per week with dsRNA molecules. Effectiveness of RNAi was tested one week after the first feeding by Real Time RT-PCR analysis. dsRNA feeding was highly effective in reducing endogenous cognate mRNA level (80% of reduction with respect to planarians fed with egg alone). Planarian specimens were sacrificed 20 days after the first feeding to analyze morphological phenotype, apoptosis, cell proliferation, epidermal cell structure and density. As expected, in planarians fed with ODC5 or ODC6 dsRNA molecules, we never observed the appearance of the morphological phenotype; activation of apoptosis, JNK upregulation, changes in cell proliferation, alteration in epidermal cell structure and density were absent as well (Supplementary Figure 6).

### Analysis of the effect of OAZ silencing, ornithine decarboxylase inhibitor DL-α DiFluoroMethylOrnithine (DFMO), and putrescine treatment, on ODC5 and ODC6 RNAi phenotype

Table 3 summarizes the effect of different dsRNA or dsRNA combinations in accordance to the way of administration and the co-treatment with other substances. In addition to what has been described in the paragraphs above, we found that OAZ dsRNA, putrescine or DFMO injection and treatment with either putrescine or DFMO, in parallel with water injection to mime punching, did not produce macroscopic phenotypic changes. The ability of *D. japonica* to take up putrescine from the medium was tested evaluating its ability to rescue DFMO-induced phenotype during regeneration, as previously described by ref. 35 (Supplementary Fig. 6I).

**Table 3:** Table summarizing the presence or absence of a macroscopic phenotype, depending on type of dsRNA molecule, mode of delivery, co-silencing and co-treatments.

TREATMENT	DELIVERY METHOD	MEDIUM	PHENOTYPE	NO PHENOTYPE
Water	injection	Water		x
*DjODC1* dsRNA	injection	Water		x
*DjODC2* dsRNA	injection	Water		x
*Smed-odc-A* dsRNA	injection	Water		x
ODC3 dsRNA	injection	Water		x
ODC4 dsRNA	injection	Water		x
ODC5 dsRNA	injection	Water	x	
ODC6 dsRNA	injection	Water	x	
ODC5 dsRNA+ODC6 dsRNA	injection	Water	x	
ODC5 dsRNA	feeding	Water		x
ODC6 dsRNA	feeding	Water		x
Water	injection	0,5 mM putrescine		x
ODC5 dsRNA	injection	0,5 mM putrescine	x	
ODC6 dsRNA	injection	0,5 mM putrescine	x	
0.5 mM putrescine	injection	Water		x
Water	injection	10 mM DFMO		x
42 mM DFMO	injection	Water		x
ODC5 dsRNA	injection	10 mM DFMO	x	
ODC6 dsRNA	injection	10 mM DFMO	x	
*DjODC5* dsRNA + *DjOAZ dsRNA*	injection	Water	x	
*DjODC6* dsRNA + *DjOAZ* dsRNA	injection	Water	x	
*DjOAZ* dsRNA	injection	water		x

The co-silencing of OAZ with either ODC5 or ODC6, did not rescue ODC5 and ODC6 RNAi macroscopic phenotype. Strikingly, the same results were obtained when putrescine or DFMO were added to the medium of ODC5 or ODC6 RNAi animals.

## Discussion

Very little is known regarding ODC function and activity in invertebrate animals. As shown by previous bioinformatics studies investigating the variability of putative AZIs and ODCs in vertebrates and invertebrates^14^, most of invertebrates have only a single copy of ODC-like genes in their genome. However, a few exceptions exist. Mosquito species like *A. aegypti*, *C. pipiens*, and *A. gambiae* were shown to be characterized by no less than 6 ODC homologs. However, as reported by the authors, only one of these homologs seems to be highly transcribed and (potentially) catalytically active, suggesting that this is the main ODC enzyme in mosquitoes. In contrast, the authors state that only one or zero ESTs were found to support the other 5 homolog expression, suggesting very low levels of transcription. Other invertebrates were found to have more than one ODC-like gene in their genome. This is the case of the chordate species *C. intestinalis* and *B. floridae*, as well as the cnidarian *H. magnipapillata*. All of them have at least one homolog with an unaltered set of the 18 key amino acids; however, no expression or functional studies have been carried to characterize the activity of ODC-like genes in these organisms. Planarians possess multiple homologs of putative ODC. The presence of substitutions in the set of 18 key amino acids essential for vertebrate ODC activity suggests that most planarian ODCs would have theoretically lost their catalytic activity, thus resembling, at least from this point of view, the vertebrate AZI. The presence of multiple copies of ODC genes in invertebrate species evolutionarily far from each other suggests that in these groups some ODC homologs could have diverged from the original decarboxylase function, especially those displaying amino acid substitutions in the key 18 residues necessary for ODC activity.

Planarian putative ODCs are expressed in post-mitotic differentiated and undifferentiated tissues but not in stem cells. This suggests that they might not be necessary for stem cells maintenance, but rather in differentiation of daughter cells.

Among the planarian ODC paralogs that we found, only one (namely *DjODC1* for *D. japonica* and *Smed-odc-A* for *S. mediterranea*) does not display substitutions in any of the 18 key amino acids, suggesting a fully functional ornithine decarboxylase activity. Surprisingly, RNAi of ODC1/A did not produce altered phenotypes, both in regenerating and intact animals. Similarly, silencing of OAZ, the direct inhibitor of ODC, does not produce any detectable macroscopic phenotype. These two findings suggest that the production of polyamines by ODC pathway could have only a secondary role in planarian homeostasis; if this would be the case, polyamines could be easily supplied directly from food, and subsequently stored within the cell. Alternatively, the presence of a parallel pathway of polyamine production (i.e. agmatinase-dependent production of putrescine), could be responsible for the lack of ODC1/A RNAi-phenotype in planarian homeostasis. Further loss of function studies for this isoform will have the potential to reveal interesting polyamine functions in planarians, as suggested by the finding that DFMO inhibits regeneration^35^.

Conversely, ODC5 and ODC6 silencing resulted in a peculiar complex phenotype, leading animal to death, which is why we focused our attention on these paralogs.

ODC5 and ODC6 RNAi leads the animal to acquire deleterious morphological alterations that were observable only when dsRNA molecules were delivered by microinjection, a condition in which a kind of chronic injury without loss of tissue is produced by the repeated punching. Indeed, the macroscopic phenotype was injection-site dependent, meaning that the animal underwent severe changes only in those regions where the injection was performed, a condition never observed in water-injected control animals.

The dramatic effect observed in epidermis morphology, as well as the upregulation of epidermal precursor markers produced by ODC5 or 6 dsRNA injection, led us to hypothesize that ODC5 and 6 could play a role in late phases of epidermal differentiation. However, epidermal aberrations were not observed in animals in which dsRNA had been delivered by feeding, suggesting that epidermal aberrations are not a consequence of impaired tissue turnover.

Head or tail regression observed in ODC5 and ODC6 RNAi-injected animals is caused by a massive activation of apoptosis in the body region in which injections have been performed. Moreover, we observed a significant increase in expression in JNK transcript only in ODC5 or 6 RNAi-injected animals. Previous studies demonstrated that JNK is only essential for responses that require the regeneration of missing tissues and it is required during the first stages of regeneration, to properly induce the expression of early wound-induced genes, the initiation of the wound-associated apoptotic peak and the controlled onset of mitosis^31^. In conclusion, we can hypothesize that silencing of ODC5 or 6 leads to no consequence if the animal is not injured, but causes serious morphological aberrations upon a chronic injury like injection. For example, the process of epidermal wound healing upon continuous punching may be impaired, making the animal to improperly perceive it as absence of tissue. This, in turn, might lead to the activation of an unnecessary regenerative response^36^, starting with the induction of apoptosis^37^, and followed by the appearance of the phenotype. Despite this appealing hypothesis, we cannot exclude the existence of other reasons that might be at the basis of differences between fed and injected organisms. For example in fed organisms, a higher tissue turn-over rate may counteract ODC5/6 RNAi effect in epidermal differentiation.

Previous studies showed a rescue of the phenotype produced by DFMO when the medium was supplied with putrescine^35^. After silencing of ODC5 and 6, if RNAi phenotype had been the consequence of reduction in polyamine synthesis, it should have been rescued by the addition of putrescine to the planarian medium or injection of this molecule in the gut. As this was not the case, we suggest that the phenotype cannot be ascribed to a putrescine production-dependent role for these proteins. This possibility is also confirmed by the inability of DFMO treatment or injection to mime ODC5 or 6 RNAi phenotype. These observations also allow us to exclude the possibility that ODC5 and 6 behave as an AZI protein, in rescuing an enzymatically active paralog (i.e. ODCA or 1) from OAZ-dependent degradation. A further hypothesis to be tested is the possibility that ODC5 and 6 form heterodimers with an enzymatically active paralog and inactivate it, thus functioning as ornithine decarboxylase inhibitors. In this case, the consequence of RNAi should be an increase in putrescine concentration that might be the cause of the phenotype. However, planarian raised in excess of putrescine and injected with water to mime the punching or directly injected with putrescine, did not develop the phenotype and the treatment of ODC5 or 6 RNAi animals with DFMO did not rescue the phenotype. Considering the numerous reports on avid polyamine uptake systems in Eukarya^4, 28–41^ and our evidence about the ability of planarian species used in this paper to take up putrescine from the medium, a very conceivable hypothesis is that ODC5 and 6 proteins play an additional polyamine-unrelated function, the loss of which produces the aberrant wound response phenotype displayed by RNAi-treated animals.

In both *S*. *mediterranea* and *D. japonica* species, ODC5 and 6 are expressed in epidermal-committed neoblast progeny cells, and their almost complete co-localization indicates that these two isoforms are expressed in the same cells. This striking result can account for either a sub-cellular compartmentalization of the two enzymes within the cell (cytoplasmic and nuclear localization for ODC has been indeed demonstrated; see 42 and references therein), or a completely new condition in which they form a heterodimer. In support of the latter hypothesis, we found that single ODC5 or ODC6 RNAi and co-silencing of the two isoforms produced the same phenotype, at least for the parameters considered in this paper. Further studies will be necessary to elucidate whether these two isoforms may interact with each other. However, if this is the case, given that ODC5 and ODC6 show complementary aminoacidic substitutions in the two key positions for catalytic activity, this would lead to the formation of two differentially active catalytic pockets, with one of them bearing both loss-of-function substitution of Lys69 (N-terminus of ODC6) and Cys360 (C-terminus of ODC5) and the other one bearing a fully-functional active site (N-terminus of ODC5 and C-terminus of ODC6). A previous study demonstrated that when mutations in Lys69 and Cys360 are introduced in two different ODC cDNAs, the catalytic activity of the dimer is maintained, yet performed by only one functional active site^43^.

The discrepancy between the phenotypes observed in ODC5/6 RNAi animals and those described for DFMO by 35, led us hypothesize that ODC5/6 might have functions, additional to the putatively halved decarboxylase activity, located in a domain that has nothing to do with ornithine decarboxylation. In fact, when using the inhibitor, the protein is still present in the cell, yet inhibited to perform decarboxylation; in this case, if a particular domain is involved in performing another function, this would be unlikely to be disrupted. Conversely, with RNAi the whole protein is depleted from the cell, leading to the absence of both decarboxylase domain and the domain supposedly involved in another activity; only the actual absence of the latter would lead to the appearance of the polyamine-unrelated phenotype. Future studies would be of great interest to elucidate these putatively additional, polyamine-independent roles of planarian ODC-like genes.

## Methods

### Animals, treatments, regeneration and morphometric analysis of blastema size

Planarians used in this work belong to the species *Dugesia japonica*, asexual strain GI^44^ and to an asexual strain of *Schmidtea mediterranea* (a kind gift of Professor Renata Batistoni, University of Pisa). Additional information on planarian rearing can be found in Supplementary methods. Regenerating fragments were obtained by transection between auricles and pharynx (in the planarian neck) or immediately below the pharynx. For morphometric analysis, animals were cut at the level of the neck, killed 4 days later in 2% hydrochloric acid for 5 min at 4 °C, and then fixed in 100% ethanol. For additional information, please see Supplementary methods.

For irradiation experiments, intact planarians were exposed to 30 Gray (Gy) (with uncertainty of ±2%) single-dose of hard X-rays using a 6 MV beam of a VARIAN Medical System accelerator for radiotherapy at the dose rate of 6 Gy/min. Putrescine hydrochloride was purchased by Sigma and diluted at 1 M in distilled water. Putrescine treatment was performed at 0.5 mM final concentration. DFMO was purchased from Cayman Chemicals, diluted at 42 mM in planarian water, and used at a final concentration of 10 mM. DFMO and putrescine media, or clean water for controls, were changed every second day. In some experiments 0.5 mM putrescine or 42 mM DFMO were directly injected in the planarian gut every two days.

### Identification of *D. japonica* and *S. mediterranea* ODC sequences and in silico analysis

Short sequences of *DjODC1*, *DjODC2* were retrieved from a DGE library containing differentially expressed TAGs obtained by comparison of a wild-type *D. japonica* DGE library and a 5 Gy-treated animal DGE library during the repopulation process that occurs after low-dose X-ray treatment (unpublished data). For the analysis, each transcript was compared to nucleotide databases (“nucleotide collection” database and “expressed sequence tags” database) by one-against-all alignments on BLAST (blastn function). *DjODC1*, *DjODC2* sequences met at least one perfect-matching transcript, and this allowed us the expand their nucleotide sequences. In addition, imperfect matches led us to find other nucleotide sequences, similar but not identical to those of *DjODC1* and *DjODC2*. These transcripts, were named *DjODC3*, *DjODC4*, *DjODC5* and *DjODC6*, and their sequences are significantly different from each other, at both nucleotide and protein level; their nucleotide sequences were expanded using blastn, as described for *DjODC1* and *DjODC2*. In some cases, nucleotide sequences were fully expanded by comparison with a transcriptome assembled from sequence reads obtained from wild-type *D. japonica* ^45^.

*S. mediterranea* ODC homologous sequences, were obtained by searching the *Schmidtea mediterranea* genome database (SmedGD)^25^ and the Planmine database^26^.

*Dugesia japonica* homologs of *Smed-NB22*.*11.e*, *Smed-agat-2*, *Smed-Collagen*, were obtained by BLAST analysis using as input *S. mediterranea* nucleotide sequences. Blastn and tblastx search were undertaken in *D. japonica* nucleotide collections/EST databases, and the transcripts with the highest similarity with those of *S. mediterranea* were taken, and called *DjNB22.11.e* (IAAB01021249.1, e-value: 2e-45 nucleotide identity 71%), *DjAGAT2* (IAAB01039299.1, e-value: 3e-52, nucleotide identity 85%), and *DjCollagen* (IAAB01067699.1, e value: 0.0, nucleotide identity 81%) respectively.

### RNA extraction, cDNA production, Real-Time RT-PCR

Planarian RNA was extracted and amplified by Real Time RT-PCR. Transcript levels of the following genes were measured: *DjODC1*, *DjODC2*, *DjODC3*, *DjODC4*, *DjODC5*, *DjODC6*, *DjPiwiA*, *Smedwi1*, *DjNB.21.11.e*, *DjMcm2*, *DjAGAT*, *Dj-mhc-b*, *2 Smed-NB.21.11.e*, *Smed-agat-1*, *Smed-odc-3*, *Smed-odc-4*, *Smed-odc-5*, *Smed-odc-6*, *Smed-ca*, *Smed-pc-2*, *Smed-collagen*, *Smed-mat*, *Smed-Vim-1*, *Smed-zfp-1*, *Smed-JNK*. The expression level of *Dj18s* and *DjEF2* was used as internal reference for experiments on *D.japonica* RNA; the stability of both genes was checked in representative samples from all tested conditions following directions described by Silver and coworkers^46^. *Smed-mat* and *Smed-luc-7* were used as internal reference for experiments on *S. mediterranea* RNA. In this last case *Smed-mat* and *Smed-luc-7* were chosen among a list of 5 five putative reference genes, tested in representative samples from all tested experimental conditions, following directions described by Silver and coworkers^46^. Additional information on RNA extraction and PCR conditions, is provided in Supplementary methods.

### Analysis of mitosis

Mitotic analysis was performed using the anti-phosphorylated histone-H3 (H3P) antibody (Upstate), both by Western blot and whole-mount immunofluorescence. For Western blot analysis, proteins were obtained by homogenizing 4 planarians per sample in lysis buffer (Tris-HCl 20 mM pH 7.4, 150 mM NaCl, 1 mM DTT, 5 mM EDTA, 5 mM EGTA, 0.1% TRITON X-100). Two independent samples were processed for each experimental condition. For a detailed protocol, please see Supplementary methods.

For immunofluorescence analysis, planarians were treated the same as for *in situ* hybridization (see below), and then incubated with 1:500 dilution of rabbit anti-H3P antibody. After several washes, specimens were incubated in alexafluor 488 anti-rabbit secondary antibody, and scanned under a Leica TCS SP8 confocal microscope. For a detailed protocol, please see Supplementary methods.

### RNAi experiments

Double-stranded RNAs (dsRNAs) of *DjODC1*, *DjODC2*, *DjODC3*, *DjODC4*, *DjODC5*, *DjODC6*, *DjOAZ*, *Smed-odc-A*, *Smed-odc-3*, *Smed-odc-4*, *Smed-odc-5*, *Smed-odc-6*, *Smed-zfp-1*, were produced by *in vitro* transcription of a template, obtained by RT-PCR on wild-type planarian cDNA, with T7 promoter adapted primers (Table S3). RNA interference (RNAi) was performed by dsRNA injection with the Nanoject Microinjectior (Drummond), or by the feeding procedure described by Rouhana and colleagues^47^. Specific downregulation of the corresponding cognate mRNA, was tested by Real Time RT-PCR 7 days after the first injection or feeding, for each dsRNA molecule. For a detailed protocol, please see Supplementary methods.

### *In situ* hybridization

DNA templates for, *Djinnexin1*, *DjPiwiA*, *DjNB.21.11.e*, *DjAGAT2*, *DjODC1-6*, *Smed-odc-5* and *6*, *DjOAZ* and *DjCollagen* were prepared by RT-PCR from wild-type planarian cDNA, using T7 promoter adapted primers, as described in Table S3. *DjSyt* DNA template was obtained as described by Salvetti and colleagues^48^. *DjInnexin10* DNA template was a kind gift of Professor Batistoni (University of Pisa). Purified amplification products were in vitro transcribed in the presence of DIG-labelling mix (Roche) to obtain digoxigenin (DIG)-labeled RNA probes or in the presence of fluorescein-labelling mix (Roche) to obtain fluorescein-labeled RNA probes. For *D. japonica* colorimetric *in situ* hybridization was performed according to Rossi and colleagues^49^. At least ten specimens were tested for each probe. For a detailed protocol please see Supplementary methods. Double fluorescent *in situ* hybridization in *S. mediterranea*, was performed according to King and Newmark^50^.

### Histological analysis

For histological examination of phenotypes, planarians were killed in 2% hydrochloric acid as described above, and fixed in 2% formaldehyde 0.2% saturated aqueous picric acid in 5/8 Holtfreter for 30 minutes at 4 °C. Specimens were then post-fixed in 2% formaldehyde, 0.1% glutaraldehyde, 0.2% saturated aqueous picric acid in 5/8 Holtfreter for 60 minutes at 4 °C. After embedding in paraffin, 6 μm thick slices were stained with Sirius red and fast green. For a detailed protocol, please see Supplementary methods. Slides were analyzed under a Zeiss Axioplan microscope. Five animals were analyzed for each experimental condition. For morphometric analysis of epidermis, 5 sections per animal were analyzed, and the thickness of both ventral and dorsal epidermis was measured in at least 6 different points per section.

### Ultrastructural microscopy

Planarians were killed in 2.5% glutaraldehyde in 0,1 M cacodylate buffer pH 7.2 for 2 hours at 4 °C, after washing in cacodylate buffer specimens were post-fixed in 0,1 M osmium tetroxide in cacodylate buffer for 2 h at room temperature. After washing in cacodylate buffer, samples were dehydrated by a graded series of ethanol and then embedded in epoxidic resin. Ultrathin sections were placed on formvar carbon grids, stained with uranyl acetate and lead citrate and analyzed under a Jeol electron microscope.

### TUNEL analysis

TUNEL assay was performed in *S. mediterranea* as described by Pellettieri and colleagues^36^ and Tu and colleagues^35^. In *D. japonica*, the assay was performed with a unique modification, namely the pre-treatment of planarians with 2% HCl / 5/8 Holtfreter solution, instead of N-acetylcysteine/PBS solution. Specimens were mounted in 80% glycerol, and scanned under the Leica TCS SP8 confocal microscope. Three planarians were analyzed for each experimental condition. A single composite image was taken for each specimen. The number of apoptotic cells was evaluated using the find *maxima* option of the Image J software.

### Propidium iodide staining and analysis of epidermis nuclear density

Planarians were fixed, bleached, rehydrated as for *in situ* hybridization and incubated for 30 minutes in 0.5 mg/ml RNAse A in PBS. Specimens were then stained with 2 µg/ml propidium iodide over night at 4 °C. After a quick wash in PBS, specimens were mounted in 80% glycerol and scanned under the Leica TCS SP8 confocal microscope. Three planarians were analyzed for each experimental condition, and composite images of epidermal nuclei were taken for both the head and tail region of each animal. The number of nuclei was counted manually in each picture.

### Statistical analysis

For comparison of blastema size a visual inspection was performed to confirm a normal distribution. Unpaired student T-test was applied to evaluate statistical significance (p < 0.05) in each single experiment, considering as matrix 1 the fifteen animals of control and matrix 2 the fifteen animals of each experimental class.

For Real Time RT-PCR, epidermal thickness and epidermal nuclei density, the non-parametric U-Mann-Whitney test was applied to evaluate statistically significant differences (p < 0.05).

## Electronic supplementary material

Supplementary information
