# Supplementary material for: Putrescine independent wound response phenotype is produced by ODC-like RNAi in planarians
Source: Sci Rep. 2017 Aug 29;7:9736. doi: 10.1038/s41598-017-09567-6 (PMC5574924; doi:10.1038/s41598-017-09567-6)
Supplement: Supplementary file 1 — Supplementary information [file 41598_2017_9567_MOESM1_ESM.pdf]

Putrescine independent wound response phenotype is produced by ODC-like RNAi in planarians

Lucia Cassella[1], Alessandra Salvetti[1], Paola Iacopetti[1], Chiara Ippolito[1], Claudio Ghezzani[1],

Gregory Gimenez[2], Eric Ghigo[3], Leonardo Rossi[1]*

Supplementary information index

Supplementary figure 1	pg 2
Supplementary figure 2	pg 3
Supplementary figure 3	pg 4
Supplementary figure 4	pg 5
Supplementary figure 5	pg 7
Supplementary figure 6	pg 8
Supplementary table 1	pg 10
Supplementary table 2	pg 11
Supplementary table 3	pg 12
Supplementary methods	pg 14

Supplementary figure 1

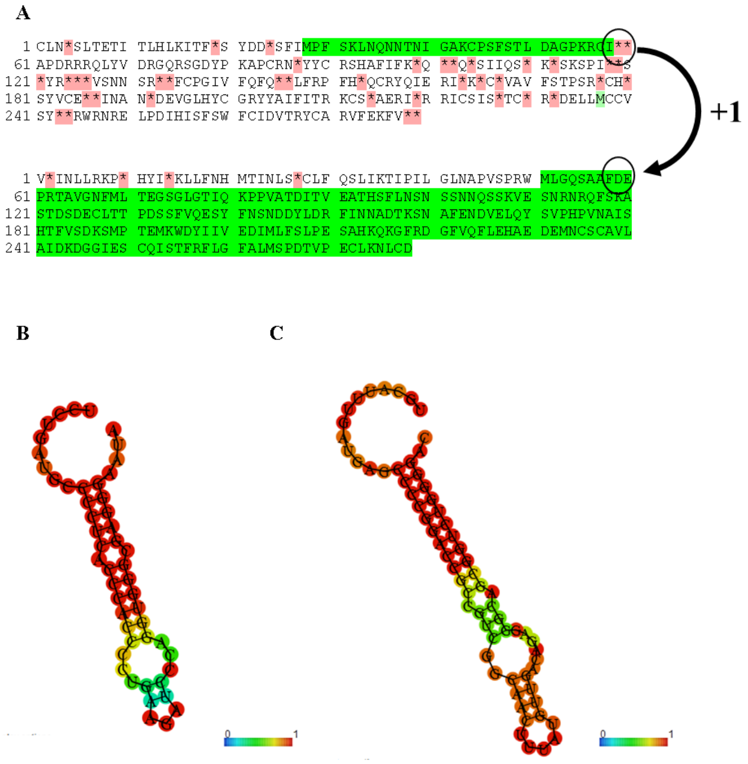

Fig. S1: *In-silico* analysis of the putative *DjOAZ* ORFs. (A) ORF1 (upper, green labelled) and ORF2 (lower, green labelled) of *D. japonica* antizyme. Circles indicate the +1 frameshift site. B-C: Antizyme secondary structure. In B is represented the secondary structure of human OAZ hairpin with a minimum free energy of -21,70 kcal/mol. In C the putative *D. japonica* OAZ hairpin is shown, arranged on a longer sequence than the human one. The structures, obtained by using the vienna RNAfold software, are colored by base-pairing probabilities (0=blue; 1=red). For unpaired regions the color denotes the probability of being unpaired. Skipped STOP codons are indicated in blue.

Supplementary figure 2

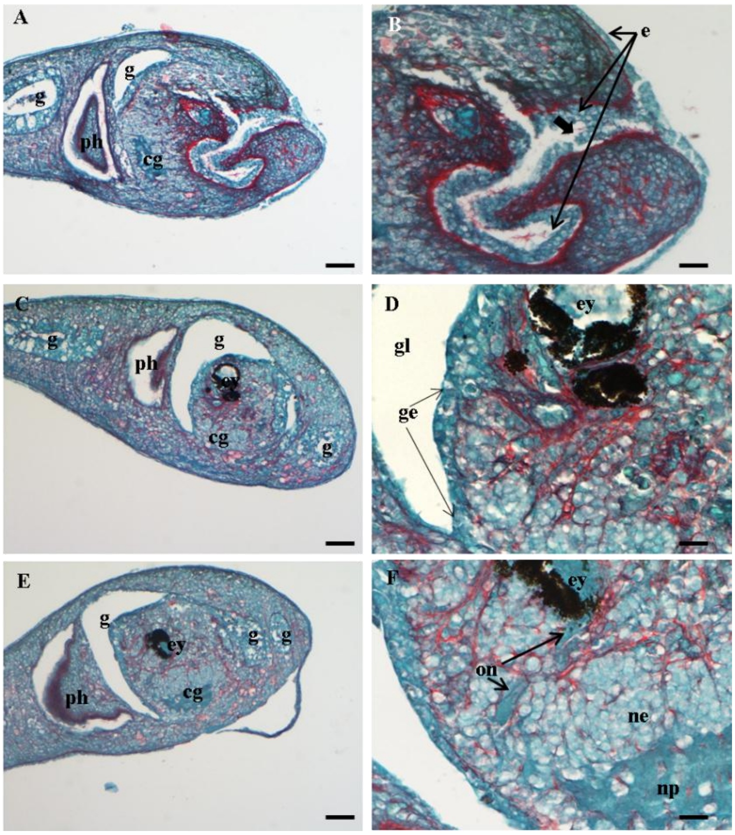

Fig. S2: Histological analysis of a representative blemmy phenotype**.** (A) A longitudinal tissue section showing the region in which the head is retracted into the posterior body part. Anterior is on the right. g: gut; ph: pharynx; cg: cephalic ganglia. (B) Magnification of A, in which the dynamical movement of retraction can be deduced by the vestigial remainings of epidermis over the red-stained layer of basal lamina, showing two prominent tissue patches generated from the invagination process. The thin duct through which the head is retracted is indicated by a thick black arrow. Thin black arrows indicate the epidermis. e: epidermis. (C) Longitudinal tissue section showing a globose head, surrounded by the anterior gut branch. Anterior is on the right. g: gut; ph: pharynx; cg: cephalic ganglia; ey: eye. (D) Magnification of C, showing the gut epithelium (ge) surrounding the retracted head. Eyes projecting toward the brain ganglia are still visible in the retracted head. gl: gut lumen. (E) Longitudinal tissue section showing the anatomical organization of the roundish retracted head. Anterior is on the right. g: gut; ph: pharynx; cg: cephalic ganglia; ey: eye. (F) Magnification of E, showing eye photoreceptors(ey) projecting through the optic nerve (on) toward the cephalic ganglia, in which a thick mass of neurons (ne) and a neuropilus (np) can be recognized. Scale bars correspond to 440 μm in A,C,E and to 70 μm in B, D, F.

Supplementary figure 3

A

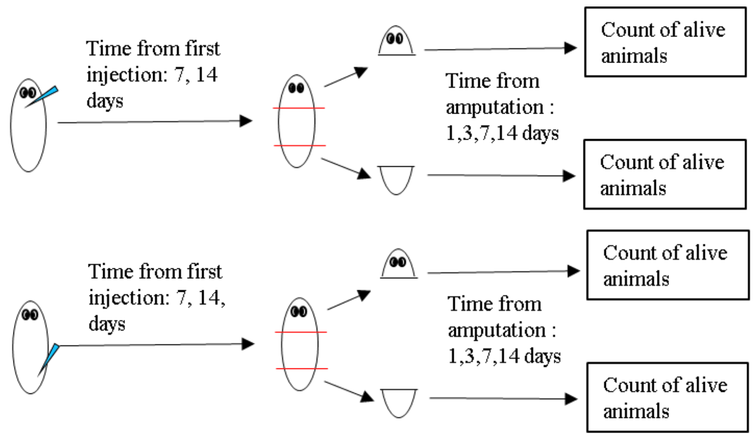

B

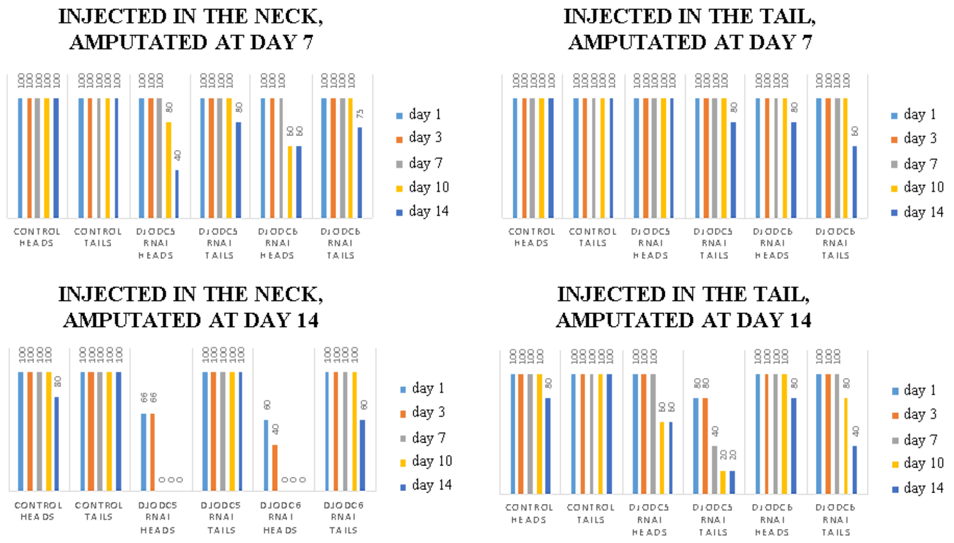

Fig. S3: Analysis of regenerating fragment viability after *DjODC5* or *6* dsRNA molecules injections. (A) Scheme depicting the experimental flow. Animals were divided into two groups, depending on whether the injection was performed anterior or posterior to the pharynx. Each group was subsequently cut into 3 pieces at different time points (7 or 14 days after the first injection). (B) Percentage of head or tail survival was measured at 1, 3, 7, 10 and 14 days post amputation in both groups. Legends on the right indicate days of regeneration.

Supplementary figure 4

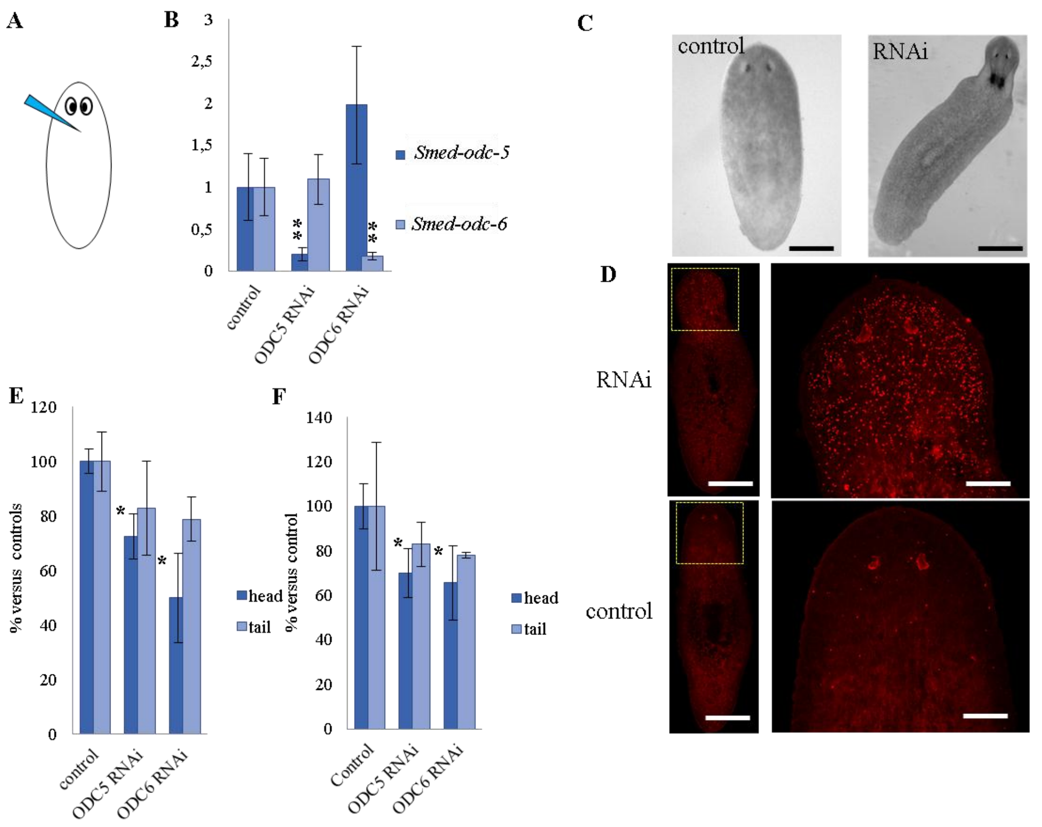

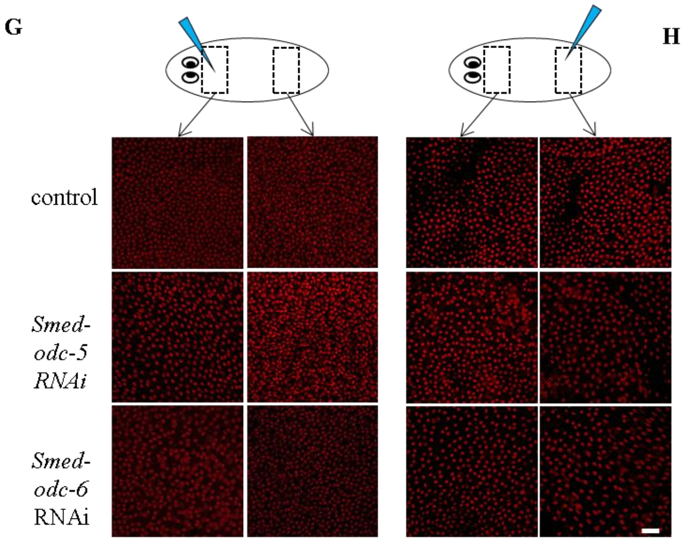

H

Folds vs control

Tissue markers	*Smed-odc-5* RNAi	*Smed-odc-6* RNAi
Smedwi1 (neoblasts)	1.48±0.05	1.37±0.07
Smed-NB.21.11.e (early zNeoblast progeny)	**3.46±0.6**	**2.5±0.14**
Smed-agat-1 (late zNeoblast progeny)	1.88±0.6	1.65±0.42
Smed-Collagen (muscle)	0.8±0.007	1.02±0.01
Smed-ca (protonephridia)		
Smed-pc2 (Central nervous system)	0.89±0.05	0.99±0.01
Smed-mat (gut)	0.98±0.02	1±0.03

Fig. S4: Effects of *Smed-odc-5* and *Smed-odc-6* dsRNA molecules injection in the neck region of *S. mediterranea.* (A) Schematic drawing of the site of injection. (B) Real Time RT PCR analysis of effectiveness and specificity of *Smed-odc-5* and *6* RNAi. Each bar is the mean ± s.d. of three independent samples, normalized versus the correspondinging control, to which an arbitrary value of 1 was attributed. ** p<0.01. (C) Representative images of a water-injected control, and a phenotype obtained by injecting dsRNA molecules in the neck region. (D) Representative images of

apoptotic cells revealed by TUNEL assay in a water-injected control, and in a phenotype obtained by injecting dsRNA molecules in the neck region. Boxed regions are enlarged on the right. Scale bars correspond to 200 μm in C, 250 μm in D (lower magnification) and 60 μm in D (higher magnification). (E) Graph depicting the epidermis thickness evaluated in body regions close to (head) and far from (tail) the injection site. Each bar is the mean ± s.d. of five independent samples in which epidermis thickness was evaluated in 5 different sections; in each taking 6 measurements. Values were normalized versus the corresponding control, to which an arbitrary value of 100% was attributed. *$p<0.05$ (F) Graph depicting numbers of nuclei counted in different body region of RNAi animals obtained by injecting dsRNA molecules in the neck region. Each bar is the mean ± s.d. of 3 independent samples. *$p<0.05$ (G) Representative images of epidermis nuclei from *S. mediterranea* specimens stained with propidium iodide after injection with water (control), *Smed-odc-5* or *6* dsRNA molecules in either the neck or in the tail region. Images are all taken at the same magnification and scale bar corresponds to 50 μm. (H) Real time RT-PCR analysis of different tissue markers in RNAi and control animals. Each value is the mean ± s.d. of three independent samples normalized versus the corresponding control to which an arbitrary value of 1 was attributed. For analysis of *Smed-mat* expression only *Smed-luc-7* was used as reference gene. Values significantly different from controls ($p<0.01$) are indicated in red.

Supplementary figure 5

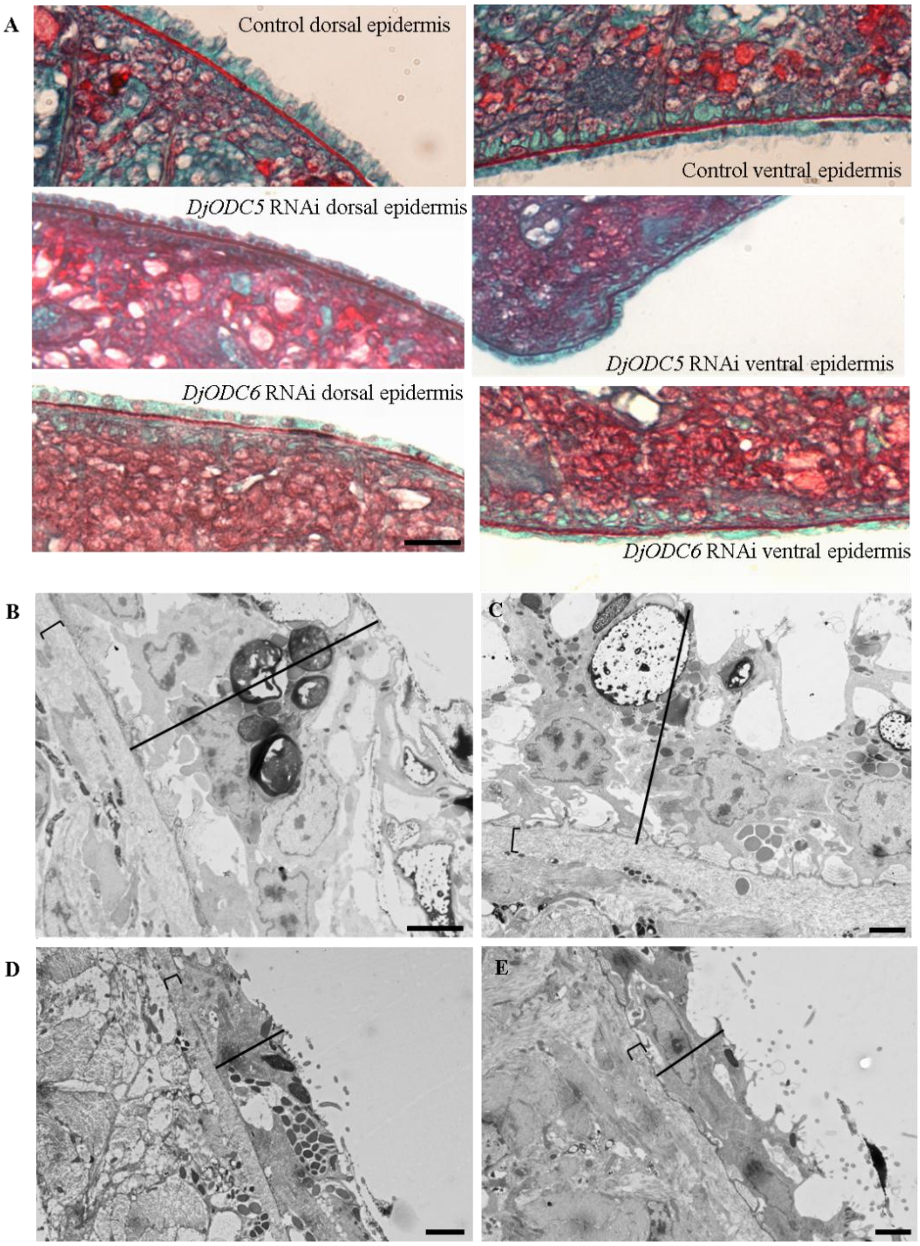

Fig S5: Histological and ultrastructural analysis of epidermis of injected planarians. (A) Representative images of transverse sections obtained from *DjODC5* dsRNA, *DjODC6* dsRNA and water injected animals. Images show epidermis of sections corresponding to the neck region, in which the injection was performed. Images were all taken at the same magnification and scale bar corresponds to 30 µm. (B) Dorsal epidermis of a water injected control from a body region far from the injection site. (C) Dorsal epidermis of a RNAi animal from a body region far from the injection site. (D) Ventral epidermis of a water injected control from a body region far from the injection site. (E) Ventral epidermis of a RNAi animal from a body region far from the injection site. The thickness of the epidermis is indicated with a black line. Square brackets indicate the basal

lamina. Scale bars correspond to 2 μm. Ultrastructural observations were performed in three independent experiments in which we analyzed several ultrathin sections of two animals per experimental class.

Supplementary figure 6

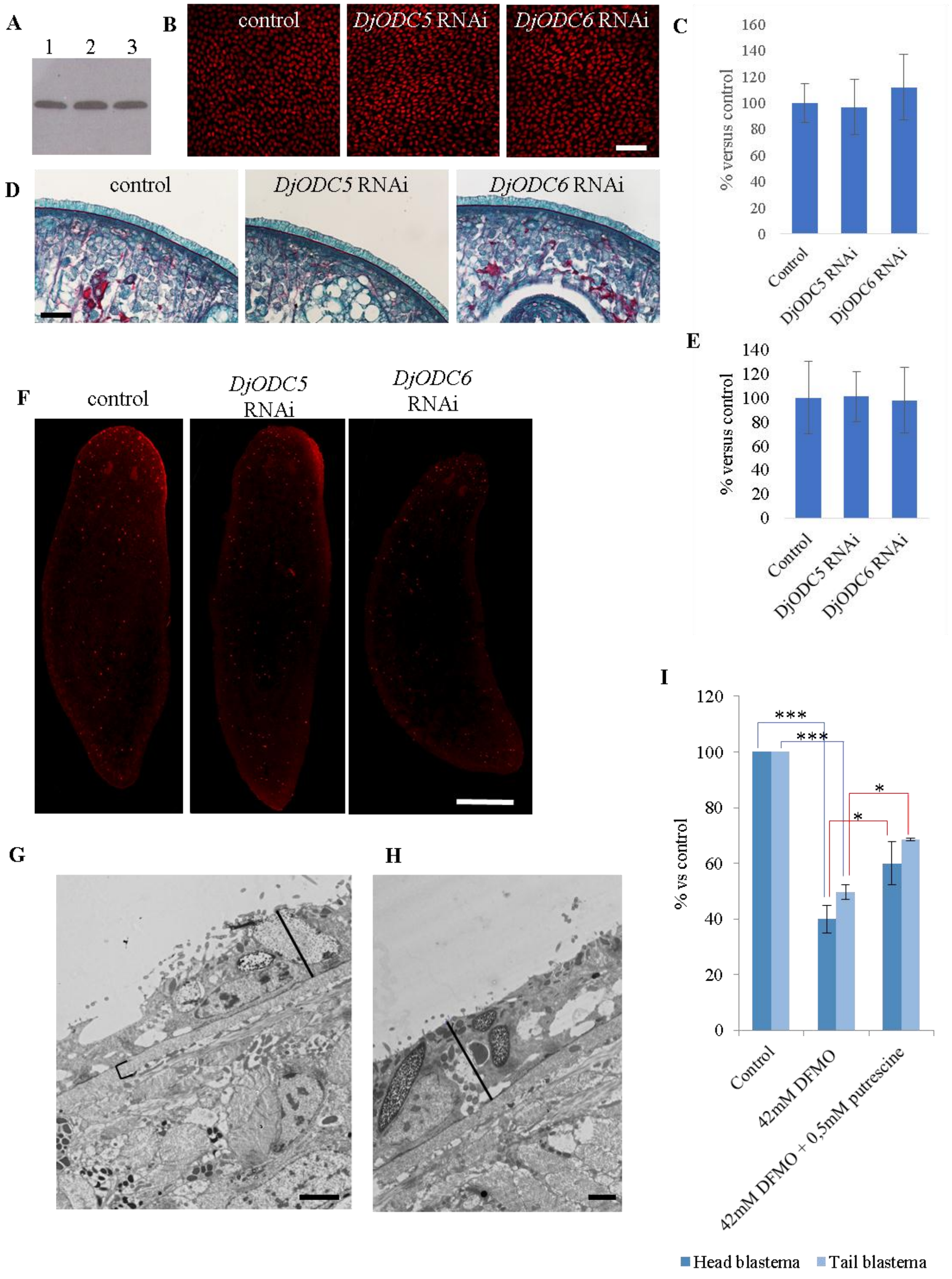

Fig. S6: Analysis of the effects produced by dsODC RNA feeding. (A) Representative Western blot showing a single cross-reactive band for phospho histone H3 in control and RNAi planarians. (B) Representative images of epidermal nuclei, stained with propidium iodide in RNAi and control planarians. Images were all taken at the same magnification and scale bar corresponds to 55 μm. (C) Graph depicting numbers of nuclei counted in RNAi and control animals. Each bar is the mean ± s.d. of 3 independent samples. (D) Representative images of sirius red stained transverse planarian sections obtained from RNAi and control animals. Images were all taken at the same magnification and scale bar corresponds to 45 μm. (E) Graph depicting the epidermis thickness evaluated in the body region close to the injection site. Each bar is the mean ± s.d. of 5 independent samples in which epidermis thickness was evaluated in 5 different sections, by taking 6 measurements in each. Values were normalized versus the corresponding control to which an arbitrary value of 100% was attributed. (F) Tunel assay in RNAi and control planarians. Tunel positive cells are visualized in red, head is toward the top. Images were all taken at the same magnification and scale bar corresponds to 250 μm. (G) Electron micrograph showing ventral epidermis ultrastructure in fed control animal. (H) Electron micrograph showing ventral epidermis ultrastructure in fed RNAi animals. Scale bars correspond to 2 μm. Ultrastructural observations were performed in three independent experiments in which we analyzed several ultrathin sections of two animals per experimental class. (I) Ability of 0.5 mM putrescine dispersed in the medium to rescue DFMO-induced phenotype during regeneration. 120 nl of 42mM DFMO were injected in the gut of intact planarians 3 hours before cutting. 40 nl were injected each following day in the gut of regenerating fragments. Medium was changed every day with a fresh 0.5 mM putrescine solution. Morphometric analysis of blastema was performed at the 4th day of regeneration. Each bar is the mean ± SD of two independent experiments each including 10 different animals. *= $p<0.05$; ***=$p<0.0001$. Unpaired student T-test was applied to evaluate statistical significance in each independent experiment considering as matrix 1 the ten DFMO injected fragments and as matrix 2 the ten control fragments or the ten DFMO injected-, putrescine treated- fragments.

SUPPLEMETARY TABLE 1

A) Blastp analysis of identified sequences of *D. japonica* and *S.mediterranea***.**

	**ID**	**Best Human Blast Hit**	**Best Eukarya Blast Hit (SwissProt)**
**DjODC1**	FY948176.1; FY958051.1; FY944481.1	Ornithine decarboxylase; Short=ODC [*Homo sapiens*] 3e-92	Ornithine decarboxylase; Short=ODC [*Mus musculus*] 6e-94
**DjODC2**	FY953982.1; FY951876.1; FY971568.1	Ornithine decarboxylase; Short=ODC [*Homo sapiens*] 5e-86	ornithine decarboxylase paralog; Short=ODC-p [*Xenopus laevis*] 7e-94
**DjODC3**	FY972384.1; BP188461.1; FY945471.1	Ornithine decarboxylase; Short=ODC [*Homo sapiens*] 2e-75	Ornithine decarboxylase 1; Short=ODC 1; Short=xODC1 [*Xenopus laevis*] 3e-80
**DjODC4**	FY932739.1; FY953652.1; FY961501.1	Ornithine decarboxylase; Short=ODC [*Homo sapiens*] 3e-79	Ornithine decarboxylase 1; Short=ODC 1; Short=xODC1 [*Xenopus laevis*] 4e-81
**DjODC5**	FY933339.1	Ornithine decarboxylase; Short=ODC [*Homo sapiens*] 9e-43	ornithine decarboxylase paralog; Short=ODC-p [*Xenopus laevis*] 2e-43
**DjODC6**	FY934642.1	Ornithine decarboxylase; Short=ODC [*Homo sapiens*] 6e-41	Ornithine decarboxylase 1; Short=ODC 1; Short=xODC1 [*Xenopus laevis*] 2e-44
**SMED-ODC-A (CLASS A)**	ox_Smed_v2_03250	Ornithine decarboxylase; Short=ODC [*Homo sapiens*] 3e-82	Ornithine decarboxylase 2; Short=ODC 2 [*Xenopus laevis*] 4e-95
**SMED-ODC-3 (CLASS B)**	uc_Smed_v2_Contig42998; dd_Smed_v4_8195_0_1	Ornithine decarboxylase; Short=ODC [*Homo sapiens*] 3e-83	Ornithine decarboxylase; Short=ODC [*Mus musculus*] 9e-87
**SMED-ODC-4 (CLASS D)**	bo_Smed_v1_gi632922150; dd_Smed_v4_5493_0_1	Ornithine decarboxylase; Short=ODC [*Homo sapiens*] 3e-83	Ornithine decarboxylase 1; Short=ODC 1 [*Xenopus laevis*] 7e-86
**SMED-ODC-5 (CLASS C)**	PublishedTranscript_Smed_v1_JX010519.1; dd_Smed_v4_6130_0_1	Ornithine decarboxylase; Short=ODC [*Homo sapiens*] 6e-46	Ornithine decarboxylase 2; Short=ODC 2 [*Xenopus laevis*] 1e-48
**SMED-ODC-6 (CLASS E)**	uc_Smed_v2_Contig45486; dd_Smed_v4_4916_0_1	Ornithine decarboxylase; Short=ODC [*Homo sapiens*] 9e-39	Ornithine decarboxylase 1; Short=ODC 1[*Xenopus laevis*] 9e-41

B) Comparison of *D. japonica* ODC nucleotide sequences with *S.mediterranea* ODC sequence classes**.** In bold are indicated the highest E-values**.** Both *DjODC1* and *DjODC2* found a match with class A sequences; the other *DjODCs* (3, 4, 5, 6) matched univocally with members of class B, D, C and E respectively.

	Class A	Class B	Class C	Class D	Class E
DjODC1	**0.0**	7e-11	7e-05	3e-09	0.44
DjODC2	**2e-117**	8e-08	8e-08	0.002	1e-05
DjODC3	4e-19	**0.0**	7e-04	1e-07	1.3
DjODC4	2e-11	2e-11	0.027	**0.0**	0.095
DjODC5	4e-11	5e-04	**2e-167**	0.84	0.84
DjODC6	0.025	1e-05	0.30	0.30	**2e-112**

SUPPLEMENTARY TABLE 2

S.mediterranea genomic region coding for putative ornithine decarboxylase (from SmedGD). Multialignments of both the nucleotide and the predicted protein sequences of twenty transcripts showed that some of them were partially overlapping. This led us to group them into five (A to E) classes of sequences, each of them containing sequences either identical or highly similar to members of the same class but significantly different from members of a different class. In particular, a high percentage of the twenty sequences retrieved belonged to class A, whose members showed a high degree of polymorphism.

	GENOMIC REGIONS	HOMOLOGY
Class A	>mk4.000260.08.01 class=Sequence position=v31.000260:62446..64446 (- strand)<br>>mk4.003300.00.01 class=Sequence position=v31.003300:9082..14869 (+ strand)<br>>mk4.005186.01.01 class=Sequence position=v31.005186:1985..2455 (+ strand)<br>>mk4.008099.00.01 class=Sequence position=v31.008099:9..326 (- strand)<br>>mk4.006981.03.01 class=Sequence position=v31.006981:23767..24927 (- strand)<br>>mk4.008445.00.01 class=Sequence position=v31.008445:24945..25492 (- strand)<br>>mk4.008941.00.01 class=Sequence position=v31.008941:158..3967 (+ strand)<br>>mk4.008941.00.02 class=Sequence position=v31.008941:3231..4088 (+ strand)<br>>mk4.014212.00.01 class=Sequence position=v31.014212:5639..10485 (- strand)<br>>mk4.015858.00.01 class=Sequence position=v31.015858:12681..14134 (+ strand)<br>>mk4.016468.00.01 class=Sequence position=v31.016468:9191..10554 (- strand)<br>>mk4.021206.00.01 class=Sequence position=v31.021206:9500..10089 (- strand)<br>>mk4.025019.00.01 class=Sequence position=v31.025019:9084..10189 (+ strand)	Ornithine decarboxylase-like (Aplysia Californica) $2e^{-98}$
Class B	>mk4.000699.03.01 class=Sequence position=v31.000699:40709..49929 (+ strand)	ODC1 (Saccoglossus kowalevskii) $1e^{-86}$
Class C	>mk4.001750.03.01 class=Sequence position=v31.001750:62577..67641 (+ strand)	ODC-1 (Schmidtea mediterranea) 0.0
Class D	>mk4.009657.00.01 class=Sequence position=v31.009657:11139..19236 (+ strand)<br>>mk4.012821.00.01 class=Sequence position=v31.012821:20863..24122 (+ strand)<br>>mk4.022984.00.01 class=Sequence position=v31.022984:99..339 (+ strand)	Ornithine decarboxylase-like (Aplysia Californica) $1e^{-67}$
Class E	>mk4.013057.00.01 class=Sequence position=v31.013057:24..2365 (+ strand)	Ornithine decarboxylase-like (Aplysia Californica) $3e^{-39}$

SUPPLEMENTARY TABLE 3

Primer sequences

	Probe F	Probe R	RealTime F	RealTime R	dsRNA F	dsRNA R
DjODC1	TGAACAATCCTATGGCTAATATCAA	CGGATATAATACGACTCACTATAGGGATTTTCGGCAAAGGTTCTTGTTGT	AGGTCTAAGCAGTGTTAATGGAGAT	GAACAAAATAGCGACCAGGCTCA	CGGATATAATACGACTCACTATAGGGTGAACAATCCTATGGCTAATATCAA	CGGATATAATACGACTCACTATAGGGATTTTCGGCAAAGGTTCTTGTTGT
DjODC2	ATGCCATCAAATGTCAACCAGAT	CGGATATAATACGACTCACTATAGGG CATCAACATCAACACCAAATCTAGC	AGTGGTTGTAATGAAATGGATGCT	TCCGCCTCCGATGTCAACAAG	CGGATATAATACGACTCACTATAGGGAATGTCAACCAGATCCAAAAGTAA	CGGATATAATACGACTCACTATAGGG CATCAACATCAACACCAAATCTAGC
DjODC3	GTTTCTCTCAACTCAGCACTT	CGGATATAATACGACTCACTATAGGGTCGGCGGTAACAAAATTCTTCAG	CCTACACTCTAGCCACCGAA	ATGTTCCGATTTTTACAATTCTGTCT	CGGATATAATACGACTCACTATAGGGACACTCTAGCCACCGAAATCATTG	CGGATATAATACGACTCACTATAGGG TCGGCGGTAACAAAATTCTTCAG
DjODC4	CACACATCTAAACTTGTAAGCGAGG	CGGATATAATACGACTCACTATAGGGTTGGGGACAGAATTCCATTGAAA	GTTACGAAATAATTGCCACTCCT	TTACTATTGGAGCTTTCACTAAATTC	CGGATATAATACGACTCACTATAGGGGCCACAGCAAGACGAAGAAAA	CGGATATAATACGACTCACTATAGGG TTGGGGACAGAATTCCATTGAAA
DjODC5	CAGTTGGGACGAAATTAATAATGCT	CGGATATAATACGACTCACTATAGGG AAAACAGACGCCCACTAAATT	AAATCTCATAACGGCATCGAATAAAA	AAGCATTATTAATTTCGTCCCAACT	CGGATATAATACGACTCACTATAGGGTGGGCACTTTCCCTGAAAAATA	CGGATATAATACGACTCACTATAGGG AAAACAGACGCCCACTAAATT
DjODC6	TGGATTCAGTTGTGCGTCATACAG	CGGATATAATACGACTCACTATAGGGTTCCTTGACATATGCCAGGAGTTC	GTGTCCAGCCATTTTTCGCTCATTC	CCAGCTCCCAACGATCCTAAAATA	CGGATATAATACGACTCACTATAGGGGGCTTCAGAATCAAGACAGATACAA	CGGATATAATACGACTCACTATAGGGTTCCTTGACATATGCCAGGAGTTC
DjPiwiA	CGTCTGTGTTTTCTATAAGTTCC	CGGATATAATACGACTCACTATAGGGAGTTCCCATCCAATTAAAGTAAAG	CGTCTGTGTTTTCTATAAGTTCC	ACTTTTGCTGGAATGTTGTTATTG		
DjMcm2			GGCAGGTGAAACATTGGGATCA	GGCTACCGACATTCCTTTGGT		
DjNB.21.11.e	CTGGTAAAGAAAGTGAATCTGAAGGT	CGGATATAATACGACTCACTATAGGGTCCTTTTTCACACTGCTCTACTTTTT	CTGGTAAAGAAAGTGAATCTGAAGGT	ATCTTCCTCGTCTAACTCTGCAAC		
DjAGAT2	ATGATTGACTATTGGAAACGTGGA	CGGATATAATACGACTCACTATAGGGATTGCATAGGGATCATGGAAATTCA	CATTAAAGGACCAAAACCAGCCG	GCCAACAGCAAAACAGTGACCAA		
Dj18S			TGTATGCTGGTGCTAGAGTGAAA	AGGAATAGGACGGTATCTGATTG		
DjEF2			GATCGTTGGGTCGCATTACTGG	CCAGGAAAAGTTGTTATAGTCCCAGTTT		
Dj innexin 1	CAATCCCGAAACTGCAAGAAA	CGGATATAATACGACTCACTATAGGGAGACATATTTTCTACTACCGAATCC				
Djmhc-b			CAACATCATCAACGTGAATTGG	AGCTCATTAAGTTTATCAACGG		
DjCollagen	AAAAATCCTCAAGGCACTAAAGAAG	CGGATATAATACGACTCACTATAGGGGCTGTGTCACTGTGAATTTTGAGAA				
Smedwi-1			CCTGATGATTGGAAGTTTGGGGAA	TGTTGAAGTGACTAGGGTTTCGAT		

Smed-ca			CGGAAATTGGGGCTATACGTCAAC	CGAGAGTGGAAAAGTTAGCAAAGT		
Smed-NB.21.11E PROG-1			CGTATATTGAATGCCGTCCTCAACATC	AGAGCTTTTATCCGAATCTTTTCCGT		
Smed-mat			GTGGATACTTATGGTGGTTGGGGA	TGACAAAGGAGCAGCAATACCAAT		
Smed-pc2			AGATTTCCGCCGAACGAGATAATG	CCATCTACAGTTCTTCCATCATCCG		
Smed-collagen			ATTCTGCCAATGGACATACATGGTT	AGGTGCTGTCACTTTATTCAACTTT		
Smed-odc-A			GAACTGCGATGCTCCATTTTATCAA	AGTACGGCTGGCATATAAGATACC	CGGATATAATACGACTCACTATAGGGCATCATCAATGGAATCCAATTTGCAC	CGGATATAATACGACTCACTATAGGGATAGTTTCACAAGTTCACTTTCGGT
Smed-odc-3			GCGTCCTTTTTATGCCGTGAAAT	TAGCACAGTCAAATCCTGCACCT	CGGATATAATACGACTCACTATAGGGAAGGTTTTAGAACTTTTGGCCTCA	CGGATATAATACGACTCACTATAGGGATGGCCAATATCTTTTTAGGCGA
Smed-odc-5	AGGATTCGATTGTGCTAGTTGGG	CGGATATAATACGACTCACTATAGGGATTGCGCATAAGCCTTAAATTGTT	GGAAGCTTGGGTTCAGGATTC	TGTCGAATACAGTAAACTCAACTCCA	CGGATATAATACGACTCACTATAGGGCGATTGTGCTAGTTGGGATGAAA	CGGATATAATACGACTCACTATAGGGATTGCGCATAAGCCTTAAATTGTT
Smed-odc-4			TCAACCGGATCAGCAAGTAATCA	ACTGGTCAATTCGATACCAACATT	CGGATATAATACGACTCACTATAGGGATGTTGGTATCGAATTGACCAGTTT	CGGATATAATACGACTCACTATAGGGTATGCCCACAACATCGAATTTCATT
Smed-odc-6	AAATGTCGGAGTGGAAAATTGGG	CGGATATAATACGACTCACTATAGGGACCGGGGCACATTTAATAAACAA	AAATGTCGGAGTGGAAAATTGGG	AGCGATTTTTGGATCTCGTTGAA	CGGATATAATACGACTCACTATAGGGACATGGCTTTACAATTGGCAAAGA	CGGATATAATACGACTCACTATAGGGACCGGGGCACATTTAATAAACAA
Smed-Vim-1			GGGGCGGGATTCATTCAAAAAAG	CGGAATTGAGTAGGTCACAAAGT		
Smed-luc7			GAGCTGAAGTTATTGGAAAGCTGT	CAAAACTTCAATAGGAATTTGGGTGG		
Smed-Agat-1			AAACGCTCTGTACTCCAAACTT	TTTGGTGCCGCTGTCCATTTAG		
Smed-zfp-1			AATTCCTCCAGTCTCAACTCGAT	GTGGTTCTTCTTGGTCAGATGAT	CGGATATAATACGACTCACTATAGGGACATCCAGACCAATTCTCCCAAA	CGGATATAATACGACTCACTATAGGGGAGTGAAGCTGGTGTTGCAAATG
Smed-JNK			TGCTTGTCATTGATCCACTTCA	GAGGCCCATTCACTTCATAATCT		

SUPPLEMENTARY METHODS

Planarian rearing and morphometric analysis of blastema size

Animals were kept in artificial water (for *D. japonica*: $CaCl_2$ 2.5mM; $MgSO_4$ 0.4mM; $NaHCO_3$ 0.8mM; KCl 77µM; for *S. mediterranea*: NaCl 1.6mM; $CaCl_2$ 1mM; $MgSO_4$ 1mM; Mg Cl_2 0.1mM; $NaHCO_3$ 1.2mM) at 18°C, and starved for at least 2 weeks before being used in the experiments.

For morphometric analysis of blastema size, fixed regenerating fragments were examined under a Zeiss stereomicroscope, and images were recorded with a Zeiss camera. Digital images were quantified using ImageJ software (1). Blastema area was determined for 15 regenerating animals obtained from two independent experiments. We considered as blastema area the unpigmented region below the wound epithelium; blastema boundary was manually marked by the operator in blind.

RNA extraction and Real-Time RT-PCR

RNA was obtained from 4 planarians, frozen and immediately processed by using the TRIZOL reagent (Invitrogen), following manufacturer's instructions. RNA was quantified using a Nanodrop spectrophotometer, purity was assayed by A260/A280 ratio analysis and 500 ng were reverse-transcribed into cDNA, using hexanucleotide-random-primers and Maxima Reverse Transcriptase (Thermo scientific), following manufacturer's instructions. Real-Time RT-PCR analysis was performed in 20 microliters using the PrecisionPLUS 2X qPCR mastermix SYBR (Primerdesign), and specific primers, as listed in Table S3, according to the following protocol: initial denaturation of 2 min at 95 °C, followed by 40 cycles of 15 sec at 95 °C and 60 sec at 60 °C. Analysis was carried out with the Eco Real-Time PCR System (Illumina) and EcoStudy software. Specificity was tested by melting curve analysis; three independent samples for each experimental condition were analyzed. RNA samples processed without the reverse-transcription step did not produced amplification products.

Western blot analysis of phosphorylated histone-H3

Protein concentration was measured by Bradford method. 20 µg of proteins for each sample were separated by SDS-PAGE and electrotransfered onto a nitrocellulose membrane. Membrane was incubated with a rabbit anti-phosphorylated histone-H3 antibody (UPSTATE), diluted 1:1000 in 1% skimmed milk. After incubation with a 1:16000 dilution of anti-rabbit peroxidase-conjugate secondary antibody (Biovision), detection was performed using Lumi-Light Western blotting Substrate reagents (Roche).

Immunofluorescence analysis of Anti-H3P antibody

After fixation, rehydrated specimens were permeabilized with 20 μg/ml proteinase K for 6 minutes at 37°C, post-fixed in 4% formalin and incubated overnight in the same mix used for in situ hybridization. Rehydrated specimens were washed three times in TPBS3 (1x PBS plus 0.3% triton X-100), blocked in 10% fetal bovine serum diluted in TPBS1 (1x PBS plus 0.1% triton X-100) for 2 hours at room temperature and incubated with 1:500 dilution of rabbit anti-phosphorylated histone-H3 antibody (UPSTATE) over/night at 4°C. After several washes in TPBS1 specimens were incubated in Alexafluor 488 anti-rabbit secondary antibody for 5 hours at room temperature. After extensive washing specimens were mounted in 80% glycerol and scanned under a Leica confocal microscope by optical sectioning every 2 µm. Five planarians of similar size were analyzed for each experimental condition. Number of mitosis was evaluated counting the number of positive dots in z-stack merged images by using the find maxima property of the image J software.

dsRNA synthesis and RNAi.

1 μg of DNA template was *in vitro* transcribed using the "High yield in vitro transcription kit" (Thermo scientific) following manufacturer's instruction. dsRNA was purified by phenol extraction and ethanol precipitation and quantified using a Nanodrop spectrophotometer. RNA interference (RNAi) was performed injecting dsRNA molecules (40 nl of a 3 μg/μl stock) by using the Nanoject Microinjectior (Drummond). For ODC genes, intact planarians were injected with dsRNA molecules twice a week until day of experiment. Injection was generally performed in the anterior gut branch, in some specific cases injections were performed in one of the posterior gut branches at the tail level. Planarians injected with water were used as negative controls. *Smed-zfp-1* RNAi was performed by feeding procedure as described by Rouhana and colleagues (2). Planarians were fed twice a week. Planarians fed with liver paste alone were used as negative controls. In some specific cases *DjODC5, DjODC6, Smed-odc-5, Smed-odc-6* RNAi was performed by feeding procedure using as food a paste of boiled egg yolk. In these cases, planarians fed with egg yolk alone were used as negative controls. RNAi animals were observed daily with a Wild Heerbrugg stereomicroscope.

Colorimetric in situ hybridization

D. japonica specimens were killed in 2 % hydrocloric acid for 5 min at 4 °C, fixed in Carnoy solution for 2 h at 4 °C, and post-fixed in absolute methanol at -20 °C for 1 h. Animals were then bleached in 5 % hydrogen peroxide in methanol overnight at room temperature, under a cold light. After gradual rehydration, animals were treated with 20 μg/ml proteinaseK at 37 °C for 6 min, and then post-fixed with 4 % formalin for 1 h at 4 °C. After triethanolamine/acetic anhydride treatment, planarians were hybridized for 72 h at 55 °C in hybridization mix containing 50 % formamide, 5X SSC, 0.1 mg/ml yeast RNA, 0.1 mg/ml heparin, 0.1 % tween 20, 10 mM DTT, 10 % dextran sulfate

and RNA probe(s). Optimal probe concentration was determined empirically for each probe. After several washes in 50 % formamide, 5X SSC, 0.1 % Tween-20, animals were blocked in 1 % Roche nucleic acid blocking reagent, dissolved in Buffer 1 (maleic acid buffer plus 0.1 % triton X-100) for 30 min, and then incubated overnight at 4 °C in 1:2000 dilution of anti-DIG AP conjugated antibody, (Roche). Following several washes in Buffer 1, specimens were prepared for signal detection. For colorimetric detection, specimens were rinsed in alkaline phosphatase buffer, and then incubated for 2-3 h in detection solution containing 175 µg/ml BCIP, 337 µg/ml NBT and 10% polyvinyl alcohol. Animals were then washed in tris/EDTA buffer, post-fixed in 4% formalin and then equilibrated in 80 % glycerol and analyzed under a Ziss stereomicroscope.

Sirius red and fast green staining

After fixation and gradual dehydration, animals were equilibrated in xylene and embedded in paraffin using standard routine protocols. 6 µm thick slices, obtained from embedded samples, were placed on poly-lysine coated slides and let to dry over night at 37°C. After removal of paraffin and gradual rehydration, slides were stained using sirius red and fast green according to the following protocol.

Slides were covered with staining solution (0.1% direct red 80 (197378, Sigma); 0.1% Fast green (F7252, Sigma) dissolved in saturated aqueous picric acid) for 60 minutes in a humid chamber. Slides were then washed two times for 2 minutes each in acidified distilled water (5ml of acetic acid in 1 liter of water) and then dipped 10 times in 100% ethanol and 10 times in xylene. Slides were then mounted with a xylene soluble mounting reagent.

1. Abramoff, M. D., Magelhaes, P. J., and Ram, S. J. Image processing with ImageJ. Biophot. 11, 36-42 (2004).

2. Rouhana, L. et al. RNA interference by feeding in vitro-synthesized double-stranded RNA to planarians: methodology and dynamics. Dev Dyn. 242, 718-30 (2013).
